# IDentif.AI: Rapidly optimizing combination therapy design against severe Acute Respiratory Syndrome Coronavirus 2 (SARS‐Cov‐2) with digital drug development

**DOI:** 10.1002/btm2.10196

**Published:** 2020-12-01

**Authors:** Agata Blasiak, Jhin Jieh Lim, Shirley Gek Kheng Seah, Theodore Kee, Alexandria Remus, De Hoe Chye, Pui San Wong, Lissa Hooi, Anh T. L. Truong, Nguyen Le, Conrad E. Z. Chan, Rishi Desai, Xianting Ding, Brendon J. Hanson, Edward Kai‐Hua Chow, Dean Ho

**Affiliations:** ^1^ The N.1 Institute for Health (N.1) National University of Singapore Singapore Singapore; ^2^ The Institute for Digital Medicine (WisDM), Yong Loo Lin School of Medicine National University of Singapore Singapore Singapore; ^3^ Department of Biomedical Engineering, NUS Engineering National University of Singapore Singapore Singapore; ^4^ Cancer Science Institute of Singapore National University of Singapore Singapore Singapore; ^5^ Defence Medical and Environmental Research Institute DSO National Laboratories Singapore Singapore; ^6^ Osmosis Baltimore Maryland USA; ^7^ Institute for Personalized Medicine, School of Biomedical Engineering Shanghai Jiao Tong University Shanghai China; ^8^ Department of Pharmacology, Yong Loo Lin School of Medicine National University of Singapore Singapore Singapore

**Keywords:** artificial intelligence, combinatory treatment, COVID‐19, digital medicine, drug development, drug interactions, SARS‐CoV‐2

## Abstract

The emergence of severe acute respiratory syndrome coronavirus 2 (SARS‐CoV‐2) led to multiple drug repurposing clinical trials that have yielded largely uncertain outcomes. To overcome this challenge, we used IDentif.AI, a platform that pairs experimental validation with artificial intelligence (AI) and digital drug development to rapidly pinpoint unpredictable drug interactions and optimize infectious disease combination therapy design with clinically relevant dosages. IDentif.AI was paired with a 12‐drug candidate therapy set representing over 530,000 drug combinations against the SARS‐CoV‐2 live virus collected from a patient sample. IDentif.AI pinpointed the optimal combination as remdesivir, ritonavir, and lopinavir, which was experimentally validated to mediate a 6.5‐fold enhanced efficacy over remdesivir alone. Additionally, it showed hydroxychloroquine and azithromycin to be relatively ineffective. The study was completed within 2 weeks, with a three‐order of magnitude reduction in the number of tests needed. IDentif.AI independently mirrored clinical trial outcomes to date without any data from these trials. The robustness of this digital drug development approach paired with in vitro experimentation and AI‐driven optimization suggests that IDentif.AI may be clinically actionable toward current and future outbreaks.

## 1 INTRODUCTION

Drug repurposing, or the use of approved and investigational therapies for other indications, has been a widely implemented strategy toward treating COVID‐19. Examples include clinical studies of ritonavir (RTV) and lopinavir (LPV)^1^; hydroxychloroquine (HCQ) in combination with azithromycin (AZT)^2^; favipiravir (FPV) in combination with tocilizumab (NCT04310228); remdesivir (RDV)^3^; and losartan (LST) (NCT04312009); dexamethasone (DEX) (NCT04381936), among others. In the SIMPLE trial with severe COVID‐19 patients, RDV met trial endpoints, reducing the median time to recovery from 15 to 11 days (*p* < 0.001), and has ultimately received regulatory authorization for emergency use in severe COVID‐19 patients in the United States, Singapore, Taiwan, Japan, European Union, India, and Australia.^4^ After demonstrating promising open‐label study results in China,^5^ FPV has been approved in India and Russia for treatment of mild and moderate COVID‐19 patients, with additional clinical trials (NCT04402203 and NCT04402203) have been initiated for further validation. The majority of trial outcomes are either pending or have not shown clinical benefit over standard of care (SOC) or placebo. As such, while drug repurposing enables rapid intervention against COVID‐19, thus far, it has not led to clarity with regard to how to best treat this disease.

Traditional methods for implementing combination therapy and monotherapy based on drug repurposing rely on mechanism of action (MOA)‐based drug selection and standard clinical dosing guidelines to achieve drug synergy and therapeutic efficacy. For example, a preclinical study showed that RDV as well as high‐dose chloroquine (CQ) were efficacious toward Severe acute respiratory syndrome coronavirus 2 (SARS‐CoV‐2) in vitro.^6^ While this is an established approach that has led to promising candidate therapies, many of these regimens were not able to translate their in vitro outcomes into successful clinical results. Therefore, optimal efficacy that is clinically relevant is a different objective that presents substantial challenges to traditional drug screening and repurposing methods. For example, if candidate effective drugs are given in combination at suboptimal respective doses, resulting efficacy is moderate or even absent. At the same time, the relative doses between drugs within a combination can substantially impact treatment efficacy and toxicity due to unpredictable drug interactions. Another common hurdle is that repurposed drugs in vitro demonstrate the desired antiviral activity only at the high concentrations not achievable in a human body at safe dosing regimens. Therefore, drug dosing has a critical role in identifying which drugs belong in the optimal combination in the first place, and optimizing treatment outcomes, particularly in combination therapy, ultimately relies on simultaneously selecting the right drugs at the right respective doses.^7^, ^8^ Reconciling drug–dose parameters also requires leveraging unpredictable drug interactions in order to mediate maximal efficacy of combination therapies. Unfortunately, simultaneously pinpointing these parameters is an extraordinarily complicated task. For example, a parameter space of 1 trillion (10^12^) possible combinations would be created from a pool of only 12 candidate therapies interrogated at 10 dose levels. This is an insurmountable barrier for traditional drug screening. Important studies have previously sought to leverage drug synergy interactions to predict multidrug combinations.^9^Other strategies have investigated higher order drug interactions to develop antimicrobial drug combinations.^10^ Bridging these findings with clinical validation remains a challenge due to the size of the experimental search space.

**FIGURE 1:**
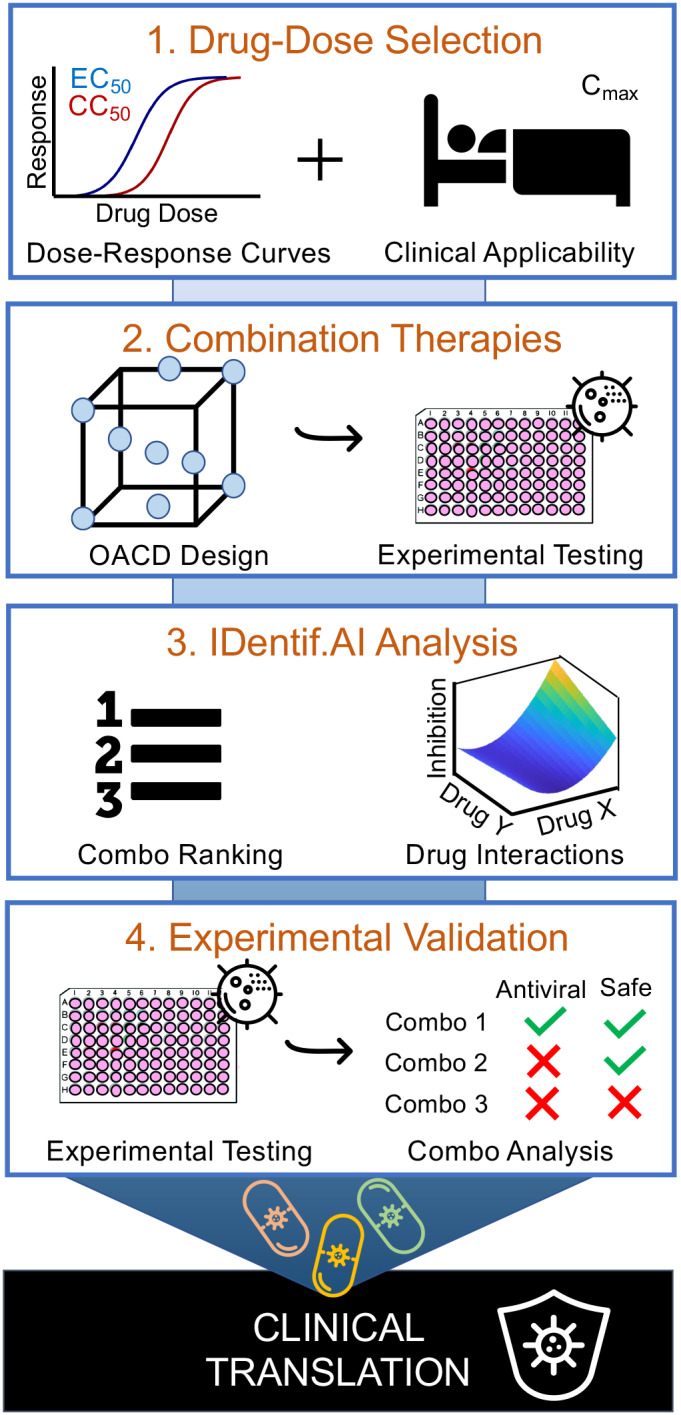
Project IDentif.AI workflow. Project IDentif.AI has four phases: (1) clinically relevant concentrations are established for each drug based on dose–response curves and maximal plasma concentration (C_max_) of clinically administered dosages, (2) combination therapies determined with an orthogonal array composite (OACD) design are experimentally tested in an in vitro, cellular infectious disease (ID) model, (3) IDentif.AI analysis of the drug dose parameter space identifies drug–drug interactions and ranks optimal, clinically relevant drug‐dosage combinations, and (4) biological validation of clinically relevant combinations designed by IDentif.AI‐designed or already in trials

In this study, we sought to overcome these challenges in developing effective combination therapies against SARS‐CoV‐2 infection using the IDentif.AI platform and an in vitro SARS‐CoV‐2 infection model with a live virus derived from a patient sample. IDentif.AI harnesses a quadratic relationship between clinically relevant therapeutic inputs (e.g., drug and dose) and biological outputs (e.g., quantifiable measurements of efficacy, safety) to experimentally pinpoint clinically relevant optimal combinations from large parameter spaces accounting for unpredictable interactions with a marked reduction in the number of required biological experiments (Figure 1).

IDentif.AI is not purely computational and does not use preexisting training datasets. Instead, it uses an orthogonally designed set of calibrating regimens and in vitro experimentation to simultaneously identify effective drugs, their unpredictable interactions and corresponding, clinically relevant doses that optimize treatment outcomes from prohibitively large drug–dose parameter spaces that cannot be reconciled by brute force drug screening.^7^, ^11^ In effect, IDentif.AI leverages these calibrating regimens to crowdsource SARS‐CoV‐2 live virus responses to experimentally drive the efficacy toward an optimal outcome. An earlier version of IDentif.AI was previously rapidly developed as a proof of concept strategy to pinpoint an optimal combination for vesicular stomatitis virus.^7^ Here, we report a clinically actionable IDentif.AI with a streamlined workflow that incorporates clinically relevant dose design, an artificial intelligence (AI)‐based strategy that prospectively and experimentally crowdsources the patient‐derived live SARS‐CoV‐2 virus to drive the optimization process, as well as a follow‐on validation process that has resulted in a ranked list of drug combinations that are simultaneously optimized for drug composition and the dose of each respective therapy. This has resulted in results that broadly and independently align with clinical trial outcomes without requiring any data from these studies, thereby resulting in a platform that can be used as a first‐line approach toward clinical decision support and therapeutic guidance with any number of additional drug options to address the COVID‐19 pandemic as well as future outbreaks. In this study, this AI‐driven digital medicine approach was applied to a 12‐drug set of candidate therapies added to a cellular infection model to pinpoint unpredictable drug interactions and clinically actionable combination therapy regimens against the live SARS‐Cov‐2 virus isolated from a nasopharyngeal swab of a patient in Singapore.^12^ The 12‐drug set included a broad spectrum of repurposed agents that were evaluated in clinical studies for treatment of COVID‐19 or were administered in conjunction with these therapies, including RDV, FPV, RTV, LPV, oseltamivir phosphate (OSV‐P), DEX, ribavirin (RBV), teicoplanin (TEC), LST, AZT, CQ, and HCQ. Noteworthy, the drugs' concentrations were clinically relevant, that is, did not exceed one‐tenth of the levels observed in the patient blood in response to standard dosing. Based on prior studies of minimal resolution experimental design, 3 clinically relevant dosing levels were employed with these 12 drugs, creating a combinatorial space of 531,000 regimens.^13^ With a three‐order of magnitude reduction in required tests, we identified a clinically actionable list of two‐, three‐, and four‐drug combinations ranked based on viral inhibition efficacy in vitro with accompanying safety data against kidney epithelial cells (Vero E6), liver epithelial cells (THLE‐2) and cardiomyocytes (AC16). The identified drugs in the combinations were all at clinically relevant concentrations, not higher than one‐tenth of the drug levels in blood in response to established clinical dosing. The top‐ranked combination was comprised of RDV, RTV, and LPV which mediated a 6.5‐fold increase in efficacy (viral inhibition %) compared to RDV alone due to an unforeseen drug interaction. Further demonstrating the clinical actionability of IDentif.AI, HCQ, and AZT combination was shown to be a relatively ineffective regimen in vitro at clinically relevant doses, mirroring recent clinical results. Importantly, the IDentif.AI‐pinpointed relative efficacy of the combinations and monotherapies at the clinically relevant doses that did not use any preexisting antiviral clinical data was independently confirmatory of many of the clinical trial endpoints to date. These outcomes, coupled with the fact that foundational precursors to IDentif.AI have been clinically validated for infectious disease, oncology, and organ transplantation human studies, support the potential application of IDentif.AI as a clinical decision support platform for the optimized design of actionable combination therapy regimens.^14^, ^15^, ^16^

## 2 RESULTS

### 2.1 Screening drug pool and experimental model

A pool of drug candidates was first chosen and evaluated for downstream IDentif.AI analysis and drug combination optimization. The pool of candidate therapies for IDentif.AI‐driven optimization contained 11 drugs that were hypothesized to inhibit SARS‐CoV‐2 viral infection via affecting: viral entry into the host cell—CQ, HCQ, AZT, LST, TEC; viral replication—RTV, LPV; viral RNA synthesis—RDV, FPV, RBV; viral release—OSV‐P.^17^, ^18^, ^19^, ^20^ To create combinations actionable within the current clinical guidelines we aimed to investigate drug interaction space between the antiviral and concomitant medications. DEX has been proposed for treating acute respiratory distress syndrome resulting from COVID‐19 (NCT04381936), LST is a common hypertension drug whose dosing should not be paused while undergoing COVID‐19 treatment.^21^ TEC is a wide spectrum antibiotic prescribed for pulmonary infections, potentially including those occurring as COVID‐19‐related complications.^22^

IDentif.AI is a dynamic optimization AI‐based platform that utilizes orthogonal array composite design (OACD), consisting of a resolution IV two‐level (drug concentrations) factorial design and a three‐level orthogonal array, to efficiently screen for influential factors and determine optimal drug‐dosage combinations within the SARS‐CoV‐2 in vitro, cellular infectious disease model. Aliasing and confounding are addressed for each independent drug's linear, bilinear (drug–drug interaction), and quadratic effects by the resolution IV design, factor screening, and deterministic nonlinear relationships.^11^, ^13^, ^23^

IDentif.AI interrogates drug–dose relationships in order to pinpoint and experimentally validate unpredictable drug interactions and the most efficacious drug combinations within defined, clinically relevant drug concentration ranges. With the ultimate goal of clinical implementation, drug–dose response in vitro experiments were performed within concentration ranges that accounted for clinically implemented concentrations and avoided clinically unrealistic drug concentrations. The viral infection model was based on virus's cytopathic effect (CPE) as a measure of the viral burden. *Z'*‐factor, the measure of the assay quality, across all experiments (*N* = 78) was 0.25, which indicated sufficient separation between the positive and negative signal bands to perform the assessment.

### 2.2 Experimental monotherapy assessment and OACD dataset construction

The drugs with and without the addition of 100 tissue culture infectious dose (TCID_50_) of SARS‐CoV‐2 virus were incubated with primate kidney cell line Vero E6 for 72 h before measuring CPE inhibition and cytotoxicity and generating the dose–response curves (Figure S1, Supporting Information). *Z'*‐factor of 0.5 for the viral plates in the monotherapies experimental set (*N* = 12) indicated suitable quality of the assay.

Only high concentrations (>1 μM) of RDV, LPV, CQ, and HCQ achieved half maximal absolute effective concentration (EC_50_) for the viral inhibition within the tested concentration ranges. High concentrations (>20 μM) of RTV, LPV and CQ led to half maximal absolute cytotoxic concentration (CC_50_) within the tested concentration ranges (Table 1). These results indicated low cellular effects of the selected monotherapies at the tested concentrations. No effect of the maximum vehicle concentration (0.1% DMSO) was detected on viral CPE inhibition or on cytotoxicity (Student's *t* test, *N* = 12, *p* > 0.05). The EC_50_ and CC_50_ of HCQ, CQ, RDV, FPV, and RBV were different from previously reported values, attributable to differences in the experimental conditions (e.g., SARS‐CoV‐2 strain, assays, incubation periods).^6^, ^24^ Regardless of the monotherapy antiviral activity, all drugs were considered for the combinatorial optimization process in order to identify possible unpredictable drug interactions that could markedly impact treatment efficacy and safety.

**TABLE 1:** Clinically relevant drug concentrations in drug combinations. Absolute half efficacy (EC_50_) and absolute half cytotoxicity (CC_50_) concentrations, and maximum plasma concentration (C_max_) and a reference for each drug. NCT number is provided for COVID‐19 clinical trials with drug dosages like those that where the basis for C_max_ selection

Drug	EC_50_ (μM)	CC_50_ (μM)	C_max_			Level 0 (μM)	Level 1 (μM)	Level 2 (μM)
			Conc. (μM)	Reference	COVID‐19 clinical trial			
RDV	1.1	>100	9	^27^	NCT04292899	0	0.81^a^	0.9^a^
FPV	>600	>600	331.83	^28^	NCT04310228	0	16.5915	33.183
RTV	>100	97	20.39	^30^	—	0	0.50975^b^	1.0195^b^
LPV	17	26	19.56	^32^	NCT04330690	0	0.978	1.956
RBV	>100	>100	17.3	^33^	NCT04276688	0	0.866	1.73
CQ	5.3	99	1.42	^34^	NCT04362332	0	0.071	0.142
HCQ	6.3	>100	5.6	^35^	NCT04261517	0	0.28	0.56
AZT	>100	>100	0.32	^36^	NCT04329832	0	0.016	0.032
OSV‐P	>10	>10	0.18	^37^	NCT04255017	0	0.009	0.018
LST	>100	>100	0.43	^41^	NCT04335123	0	0.01075^b^	0.0215^b^
TEC	>50	>50	20.475	^42^	—	0	0.511875^b^	1.02375^b^
DEX	>100	>100	0.63	^43^	—	0	0.0315	0.063

*Note*: Concentration Levels 1 and 2 were based on: a) absolute EC10 and absolute EC20 for RDV and b) 2.5 and 5% of C_max_ for RTV, LST, and TEC; and 5 and 10% of C_max_ for the rest of the drugs.

Abbreviations: AZT, azithromycin; CQ, chloroquine; DEX, dexamethasone; FPV, favipiravir; HCQ, hydroxychloroquine; LPV, lopinavir; LST, losartan; OSV‐P, oseltamivir phosphate; RBV, ribavirin; RDV, remdesivir; RTV, ritonavir; TEC, teicoplanin.

Accounting for a common source of failure in translating in vitro results to clinical trials, the high ratio of EC_50_ to maximum plasma concentration (C_max_) achieved in the human body,^25^ C_max_ was included as a crucial consideration for selecting drug concentrations at Levels 1 and 2 for each drug that ensure none of the drugs were overrepresented in relation to other drugs and to human pharmacokinetics (Table 1). Additionally, evidence has emerged that SARS‐CoV‐2 infection causes pathology of the vascular system and may require a treatment maintaining sustained drug level in the blood.^26^, ^27^

We examined C_max_ for each drug as specified in clinical data after reaching a steady state at an established dosing regimen given to a population without drug metabolism impairment and at dosing regimens listed on a drug label specified by a national regulatory body (Food and Drug Administration [FDA] in the United States; European Medicines Agency in European Union; and Pharmaceuticals and Medical Devices Agency [PMDA] in Japan) or literature. When the C_max_ information was available for multiple dosages, the dosage tested in current clinical trials for COVID‐19 was included in the considerations for selecting C_max_. RDV administered at 200 mg intravenously, had a reported C_max_ of 9.0 μM on Day 1.^28^ High dose FPV, administered at 2000/400/400 mg on Day 1 and 400 mg thrice daily (tid) for Days 2–6, had a reported C_max_ of 52.13 mg/L on Day 6.^29^ The reported C_max_ for RTV given at a high dose of 600 mg twice daily (bid), with and without other antiviral drugs, varies between 11 and 14.7 mg/L.^30^, ^31^, ^32^ LPV requires a pharmacokinetic enhancer. When given at 400/100 mg bid LPV/RTV, reported C_max_ for LPV reaches 12.3–12.9 mg/L.^28^, ^33^ The reported C_max_ for RBV administered orally bid at a total daily dose of 800, 1000, or 1200 mg, was 4.23 mg/L at Week 4.^34^ The reported C_max_ for CQ was 0.73352 mg/L when given at an initial 450 mg dose followed by two 300 mg doses.^35^ In accordance with the FDA label, HCQ reaches a C_max_ of 2.436 mg/L after a single intravenous high dose of 310 mg.^36^ The FDA label reported steady‐state C_max_ of AZT is 0.24 mg/L at a standard once daily (qd) 250 mg dose, following a 500 mg initial dose.^37^ 75 mg OSV‐P bid or qd given to healthy and obese populations resulted in a reported C_max_ of 0.0594–0.0744 mg/L.^38^, ^39^, ^40^ LST given for 7 days at 50 mg qd has a reported C_max_ of 0.1976–0.224 mg/L.^41^, ^42^ TEC requires dosing according to therapeutic dose monitoring with the minimum effective plasma concentration of 10 mg/L.^43^ The C_max_ adapted in our calculations was 35 mg/L. A single dose of 20 mg DEX given orally is reported in the FDA label to result in a C_max_ of 0.247 mg/L.^44^

In order for IDentif.AI to determine optimized drug combinations from this 12‐drug set, 100 drug–dose combinations were generated according to OACD (Table S1, Supporting Information) and, together with drug monotherapies at concentration Levels 1 and 2, were evaluated for their antiviral and cytotoxic activity on Vero E6 cells. The upper bound (Level 2) for drug concentration selection was set as 10% C_max_ or EC_20_, whichever lower. To account for high binding levels (>97%) to human plasma protein of RTV, LST, and TEC,^31^, ^45^, ^46^ we decreased their upper concentration bound to 5% C_max_. Half of the upper bound concentrations, 5% C_max_, EC_10_ or 2.5% C_max_, guided the selection of the mid drugs concentrations (Level 1). Exclusion of the drug from the combination (concentration 0 μM), served as Level 0 (Table 1). Drug combinations' cytotoxicity was additionally tested on human cell lines: liver (THLE‐2), and cardiac myocytes (AC16). *Z'*‐factor of 0.65 for the viral plates in OACD experimental set (*N* = 24) indicated high quality of the assay. No effect of the maximum vehicle concentration (0.006% DMSO) was detected on viral CPE inhibition or on cell cytotoxicity (Wilcoxon rank‐sum test, *N* = 18, *p* > 0.05).

### 2.3 IDentif.AI analysis and clinically relevant drug combination optimization

Utilizing the single drug and OACD drug in vitro data, IDentif.AI analysis determined unforeseen drug–drug interactions and pinpointed RDV/RTV/LPV to be the most efficacious three‐drug combination at the clinically relevant doses. It was also present in all top 10 ranked four‐drug combinations. RDV/LPV was the top ranked two‐drug combination (Table 2). While RDV was identified as the most efficacious single drug at a clinically relevant dose, in line with current clinical trial outcomes, IDentif.AI analysis of the experimental data determined that the three‐drug combination of RDV/RTV/LPV is critical for achieving maximal therapeutic efficacy without increasing the doses beyond what is currently clinically established. IDentif.AI analysis allows for comparative ranking of all possible combinations and drug–drug interactions within the 12‐drug set, including analysis of regimens currently being clinically investigated but that are not observed as top ranked optimized drug combinations. Both LPV/RTV (Kaletra) and HCQ/AZT have been clinically evaluated as potential treatments against SARS‐CoV‐2 infection with discouraging outcomes. IDentif.AI analysis of our experimental data revealed that they were identified to be suboptimal—LPV/RTV ranked 1261 and HCQ/AZT ranked 5161 among all 9968 drug combinations that include up to four‐drugs, with predicted viral CPE inhibition efficacies of 23% and 2%, respectively. The aforementioned findings were based on the IDentif.AI quadratic series assessing the %Inhibition experimental data with a close proximity as indicated by adjusted *R* ^2^ of 0.898 (Table S2, Supporting Information).

**TABLE 2:** IDentif.AI top ranked combinations at clinically relevant doses. Top ranked IDentif.AI determined four‐, three‐, and two‐drug combinations at clinically relevant doses with corresponding %Inhibition and %Cytotoxicity of Vero E6, AC16, and THLE‐2. Monotherapies with corresponding %Inhibition and %Cytotoxicity Vero E6. Monotherapy experiments were run in triplicate

								IDentif.AI			
Rank	Top four‐drug combinations (concentration in μM)							%Inhibition	%Cytotoxicity	%Cytotoxicity (AC16)	%Cytotoxicity (THLE‐2)
1	RDV (0.9)	+	RTV (1.0195)	+	LPV (1.956)	+	DEX (0.063)	96.6	−0.8	−9.6	12.9
2	RDV (0.9)	+	RTV (1.0195)	+	LPV (1.956)	+	RBV (0.866)	95.6	5.2	−13.1	64.5
3	RDV (0.9)	+	RTV (1.0195)	+	LPV (1.956)	+	RBV (1.73)	94.6	−0.1	−23.5	70.4
4	RDV (0.9)	+	RTV (1.0195)	+	LPV (1.956)	+	DEX (0.0315)	88.9	0.1	−20.3	44.8
5	RDV (0.9)	+	RTV (1.0195)	+	LPV (1.956)	+	AZT (0.032)	82.4	0.6	−25.6	70.5
Rank	Top three‐drug combinations (concentration in μM)							%Inhibition	%Cytotoxicity	%Cytotoxicity (AC16)	%Cytotoxicity (THLE‐2)
1	RDV (0.9)	+	RTV (1.0195)	+	LPV (1.956)			81.2	1.0	−19.4	65.1
2	RDV (0.9)	+	RTV (0.50975)	+	LPV (1.956)			67.4	2.4	−1.9	59.9
3	RDV (0.81)	+	RTV (1.0195)	+	LPV (1.956)			66.2	0.6	−17.2	67.0
4	RDV (0.9)	+	LPV (1.956)	+	AZT (0.032)			66.2	3.6	−13.2	36.1
5	RDV (0.9)	+	LPV (1.956)	+	RBV (0.866)			64.9	8.1	13.7	43.5
Rank	Top two‐drug combinations (concentration in μM)							%Inhibition	%Cytotoxicity	%Cytotoxicity (AC16)	%Cytotoxicity (THLE‐2)
1	RDV (0.9)	+	LPV (1.956)					53.6	3.9	7.4	44.1
2	RDV (0.81)	+	LPV (1.956)					38.6	3.5	9.6	44.1
3	RDV (0.9)	+	LPV (0.978)					38.1	2.7	−17.4	54.9
4	RDV (0.9)	+	AZT (0.032)					35.3	1.2	−33.4	57.6
5	RDV (0.9)	+	RBV (0.866)					32.2	5.7	−17.5	63.3
Rank	Clinically relevant combinations (concentration in μM)							%Inhibition	%Cytotoxicity	%Cytotoxicity (AC16)	%Cytotoxicity (THLE‐2)
1261^a^	RTV (1.0195)	+	LPV (1.956)					23.2	−2.6	1.9	18.9
5161^a^	HCQ (0.28)	+	AZT (0.016)					1.9	9.6	17.5	−16.6
								Experimental			
Rank	Monotherapies (concentration in μM)							%Inhibition (mean ± propagated *SD*)		%Cytotoxicity (mean ± propagated *SD*)	
1	RDV (0.9)							21.1 ± 5.4		2.2 ± 7.1	
2	RDV (0.81)							15.5 ± 2.8		0.3 ± 7.5	
3	FPV (33.183)							1.9 ± 1.8		5.1 ± 5.6	
4	CQ (0.071)							1.4 ± 4.2		4.2 ± 9.3	
5	RTV (1.0195)							0.9 ± 1.4		0.5 ± 11.0	

*Note*: Data are shown as mean ± propagated *SD*; *N* = 3.

Abbreviations: AZT, azithromycin; CQ, chloroquine; DEX, dexamethasone; FPV, favipiravir; HCQ, hydroxychloroquine; LPV, lopinavir; OSV‐P, oseltamivir phosphate; RBV, ribavirin; RDV, remdesivir; RTV, ritonavir; TEC, teicoplanin.

^a^ Corresponds to rank of out 9968 combinations (four drugs or less).

Multiparameter IDentif.AI analysis of the experimental data allowed cytotoxicity of ranked combinations to be interrogated as well via deriving %Cytotoxicity quadratic series (Tables S3–S5, Supporting Information). In interpreting cytotoxicity results, it is important to note that %Cytotoxicity is calculated in relation to the control cell culture luminescence and as such, it has a different scale than %Inhibition, which is calculated in relation to the luminescence dynamic range, and the two values should be interpreted independently. The top ranked three‐ and four‐drug RDV‐based combinations were determined to have similar %Cytotoxicity in the Vero E6 cells as the experimentally measured single drug RDV treatments. IDentif.AI analysis also determined low %Cytotoxicity for the top three‐drug combination, RDV/RTV/LPV in the AC16 cells and higher %Cytotoxicity in the THLE‐2 cells. This IDentif.AI‐derived THLE‐2 %Cytotoxicity was predicted to decrease with the addition of DEX in the top four‐drug combination (Table 2). Outlier analysis performed for each IDentif.AI quadratic series (Figures S2–S5, Supporting Information) identified and excluded OACD combination 15 from the AC16 %Cytotoxicity data set (Figure S4, Supporting Information) and combination 46 from the THLE‐2 %Cytotoxicity data set (Figure S5, Supporting Information). These data sets were subsequently reanalyzed (Figures S6 and S7, Supporting Information). The plot of residuals against fitted values (Figure S2, upper left panel, Supporting Information) showed a tendency of points to cluster over the range of 0–50% inhibition; however, this distribution of fitted values follows that of experimental data. Additionally, the probability plots and residual histograms (Figure S2, lower panels, Supporting Information) did not reveal any obvious deviations from normality and homoscedasticity.

Taken together, in vitro data analyzed with IDentif.AI pinpointed RDV‐based treatments to likely be the most effective therapies against SARS‐CoV‐2 infections without the need to increase the drug doses beyond clinically established regimens, with RDV/RTV/LPV capable of achieving maximal efficacy with potential reductions in overall toxicity if complemented with the fourth drug.

### 2.4 Experimental validation of IDentif.AI results

**FIGURE 2:**
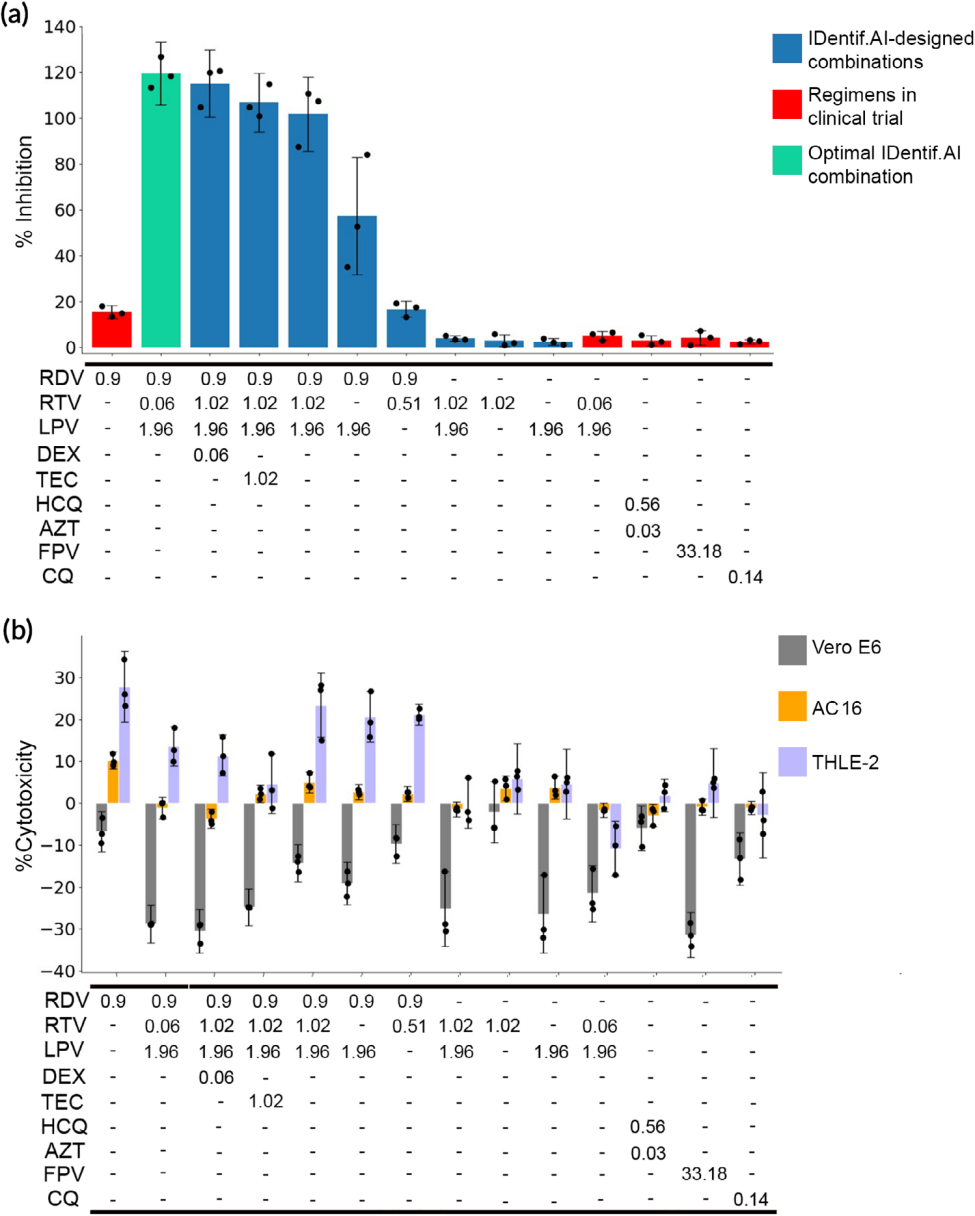
Experimental validation of the IDentif.AI‐designed at clinically relevant doses. (a) %Inhibition of the optimal IDentif.AI combination of RDV/LPV/RTV (green), IDentif.AI designed combinations (blue), and regimens in clinical trials (red). (b) %Cytotoxicity of Vero E6 (gray), AC16 (orange), and THLE‐2 (purple), of the optimal IDentif.AI combination of RDV/LPV/RTV, IDentif.AI designed combinations, and regimens in clinical trials. Data are shown as mean ± propagated *SD*; *N* = 3. Kruskal‐Wallis test detected statistically significant differences at p<0.001 for the %Inhibiton and the %Cytotoxicity groups, but the pairwise comparisons with Dunn's post hoc did not detect any statistically significant differences

Selected data points derived from the OACD experimental set via the IDentif.AI analysis were validated in the in vitro assay. *Z'*‐factor of 0.61 for the viral plates in the validation experimental set (*N* = 3) indicated high quality of the assay. Validation results were interpreted considering not only the *p*‐values, but also the logic, background knowledge and specifics of the experimental design.^47^ The cellular viral CPE inhibition experiments confirmed IDentif.AI ranking of RDV in combination with LPV and RTV as the optimal combination of the study, resulting in complete viral CPE inhibition (Figure 2(a) and Table 3) at clinically actionable concentrations. This combination resulted in a 6.5‐fold increase in efficacy compared to RDV alone. RDV at a clinically relevant concentration was confirmed as an essential driver of the antiviral efficacy in the optimized combinations, even though it mediated only moderate antiviral effect on its own. These cellular validation experiments were conducted following IDentif.AI identification of top‐ranked optimized RDV‐based drug combinations and comparative ranking of these combinations against other possible combinations.

**TABLE 3:** Experimental validation results for the IDentif.AI‐designed combinations. IDentif.AI‐designed top four‐drug combinations, probing dosing space of RTV in combinations, clinically discussed combinations for COVID‐19, top three‐drug combinations with concomitant drugs (TEC and LST), and monotherapies with corresponding %Inhibition, %Cytotoxicity, %Cytotoxicity AC16, and %Cytotoxicity THLE‐2

							Experimental (mean ± propagated *SD*)			
							%Inhibition	%Cytotoxicity	%Cytotoxicity (AC16)	%Cytotoxicity (THLE‐2)
Top four‐drug combinations (concentration in μM)										
RDV (0.9)	+	RTV (1.0195)	+	LPV (1.956)	+	DEX (0.063)	115.1 ± 14.6	−30.5 ± 5.2	−3.7 ± 2.3	11.4 ± 4.9
RDV (0.9)	+	RTV (1.0195)	+	LPV (1.956)	+	RBV (0.866)	104.5 ± 16.8	−24.9 ± 5.9	3.5 ± 2.1	14.5 ± 4.7
RTV (1.0195)	+	LPV (1.956)	+	RBV (0.866)	+	DEX (0.063)	11.3 ± 7.1	−20.8 ± 10.0	−7.9 ± 2.5	−8.7 ± 12.6
RBV (0.866)	+	HCQ (0.56)	+	OSV‐P (0.018)	+	LST (0.0215)	3.3 ± 2.0	−9.6 ± 9.2	−1.5 ± 2.2	−7.4 ± 4.8
FPV (33.183)	+	HCQ (0.56)	+	OSV‐P (0.018)	+	LST (0.0215)	4.7 ± 2.0	−13.5 ± 4.9	−3.1 ± 3.0	−9.4 ± 8.1
Probing dosing space (concentration in μM)										
RDV (0.9)	+	RTV (1.0195)	+	LPV (1.956)			101.8 ± 16.1	−14.3 ± 4.4	5.0 ± 2.5	23.4 ± 7.6
RDV (0.9)	+	RTV (0.50975)	+	LPV (1.956)			83.8 ± 25.4	−3.3 ± 4.6	0.1 ± 1.9	16.7 ± 5.0
RDV (0.9)	+	RTV (1.0195)	+	LPV (0.978)			27.4 ± 5.5	−29.6 ± 5.0	−0.8 ± 1.7	11.9 ± 4.0
RDV (0.9)	+	RTV (0.05597)	+	LPV (1.956)			119.6 ± 13.7	−28.8 ± 4.5	−1.1 ± 2.6	13.5 ± 4.7
RTV (1.0195)	+	LPV (1.956)					3.9 ± 1.1	−25.2 ± 8.9	−1.5 ± 1.8	0.0 ± 6.0
RTV (0.05597)	+	LPV (1.956)					5.2 ± 2.0	−21.5 ± 6.7	−1.7 ± 1.7	−10.8 ± 6.5
RDV (0.9)	+	LPV (1.956)					57.4 ± 25.6	−19.2 ± 5.1	2.7 ± 1.7	20.6 ± 6.0
RDV (0.9)	+	RTV (0.50975)					16.7 ± 3.5	−9.7 ± 4.6	2.2 ± 1.8	21.1 ± 2.5
Clinically discussed combinations (concentration in μM)										
HCQ (0.56)	+	AZT (0.032)					3.0 ± 2.1	−6.0 ± 5.3	−2.9 ± 2.7	1.9 ± 3.9
FPV (33.183)	+	RTV (1.0195)	+	LPV (1.956)	+	DEX (0.063)	9.8 ± 1.4	−45.8 ± 7.2	−9.8 ± 2.1	−5.8 ± 2.8
FPV (33.183)	+	RTV (1.0195)	+	LPV (1.956)			10.6 ± 3.6	−40.1 ± 6.9	−4.4 ± 2.8	11.8 ± 4.2
Top three‐drug combination with concomitant drugs (concentration in μM)										
RDV (0.9)	+	RTV (1.0195)	+	LPV (1.956)	+	TEC (1.02375)	106.9 ± 12.9	−24.8 ± 4.3	2.2 ± 2.1	4.6 ± 7.1
RDV (0.9)	+	RTV (1.0195)	+	LPV (1.956)	+	LST (0.0215)	115.4 ± 16.8	−12.6 ± 8.6	8.7 ± 1.8	26.5 ± 7.1
Monotherapy (concentration in μM)										
RDV (0.9)							15.5 ± 2.8	−6.8 ± 4.8	10.2 ± 2.0	27.8 ± 8.4
RTV (1.0195)							2.9 ± 2.5	−2.1 ± 7.3	3.6 ± 2.9	5.8 ± 8.4
LPV (1.956)							2.5 ± 1.5	−26.4 ± 9.2	3.8 ± 2.7	4.6 ± 8.4
DEX (0.063)							4.6 ± 3.5	−32.4 ± 6.4	−1.1 ± 2.5	6.5 ± 8.6
HCQ (0.56)							3.9 ± 1.5	−23.3 ± 6.4	6.8 ± 2.0	9.6 ± 14.2
FPV (33.183)							4.2 ± 3.1	−31.4 ± 5.4	−0.9 ± 2.0	4.8 ± 8.2
RBV (0.866)							0.3 ± 1.0	−14.8 ± 7.7	2.0 ± 1.5	−0.4 ± 10.1
CQ (0.142)							2.4 ± 1.0	−13.3 ± 6.2	−1.1 ± 1.6	−2.8 ± 10.2

*Note*: Data are shown as mean ± propagated *SD*; *N* = 3.

Abbreviations: AZT, azithromycin; CQ, chloroquine; DEX, dexamethasone; FPV, favipiravir; HCQ, hydroxychloroquine; LPV, lopinavir; LST, losartan; Abbreviations: AZT, azithromycin; CQ, chloroquine; DEX, dexamethasone; FPV, favipiravir; HCQ, hydroxychloroquine; LPV, lopinavir; LST, losartan; OSV‐P, oseltamivir phosphate; RBV, ribavirin; RDV, remdesivir; RTV, ritonavir; TEC, teicoplanin.

LPV and RTV are commonly administered together as RTV acts as a pharmacokinetic enhancer of LPV. Of note, the high antiviral effects of RDV/RTV/LPV were sustained when the RTV concentration was decreased 20‐fold, and the RTV/LPV concentrations reflected the standard 100/400 mg bid dosing in its clinically administered formulation (Kaletra). As such, RDV/RTV/LPV combination likely does not require increasing RTV dose beyond what is commonly used clinically and is readily clinically actionable upon an approval. RDV's cytotoxicity, also reported clinically,^28^ was not enhanced in any of the combinations (Figure 2(b)). In fact, the in vitro results suggest that RDV/RTV/LPV may suppress RDV‐induced cytotoxicity both on human cardiac myocytes and human liver cell lines. The addition of DEX to the RDV/RTV/LPV combination further reduced the cytotoxicity across all three cell lines. Glucocorticoid exposure to epithelial cells—such as liver, heart, and kidney—is known to lead to an antiapoptotic effect and the DEX‐mediated cytoprotection has been demonstrated in several cell lines.^48^, ^49^ We also observed the positive effect of DEX on cell growth in the monotherapy experimental set. It is plausible that the observed reductions in drug‐induced cytotoxicity in combinations with DEX are due to DEX‐mediated cytoprotection and requires further investigation. Noteworthy, the coadministration of DEX did not decrease the efficacy of the antiviral treatment. Importantly, dose limiting and drug exclusion experiments further deciphered the contribution of each drug toward overall RDV/RTV/LPV antiviral activity at clinically relevant concentrations. RDV was confirmed to have the greatest contribution, with LPV/RTV on its own not mediating viral CPE inhibition in vitro. High concentrations of LPV were critical to maximizing the RDV/RTV/LPV antiviral activity. While the concentration of RTV was not a critical determinant of the resulting efficacy of the combination, the presence of RTV was critical to RDV/RTV/LPV achieving maximal viral CPE inhibition in vitro, at clinically relevant concentrations. Further confirming the accuracy of IDentif.AI analysis, validation of clinically trialed treatments against COVID‐19, LPV/RTV (Kaletra),^1^ HCQ/AZT,^50^, ^51^ FPV (NCT04310228), and CQ (NCT04362332) did not induce as much viral CPE inhibition as compared to RDV alone. These data confirm that IDentif.AI can accurately reflect the unsatisfactory outcomes observed in those clinical trials, without incorporating any prior clinical data or drug mechanism assumptions as inputs.

**FIGURE 3:**
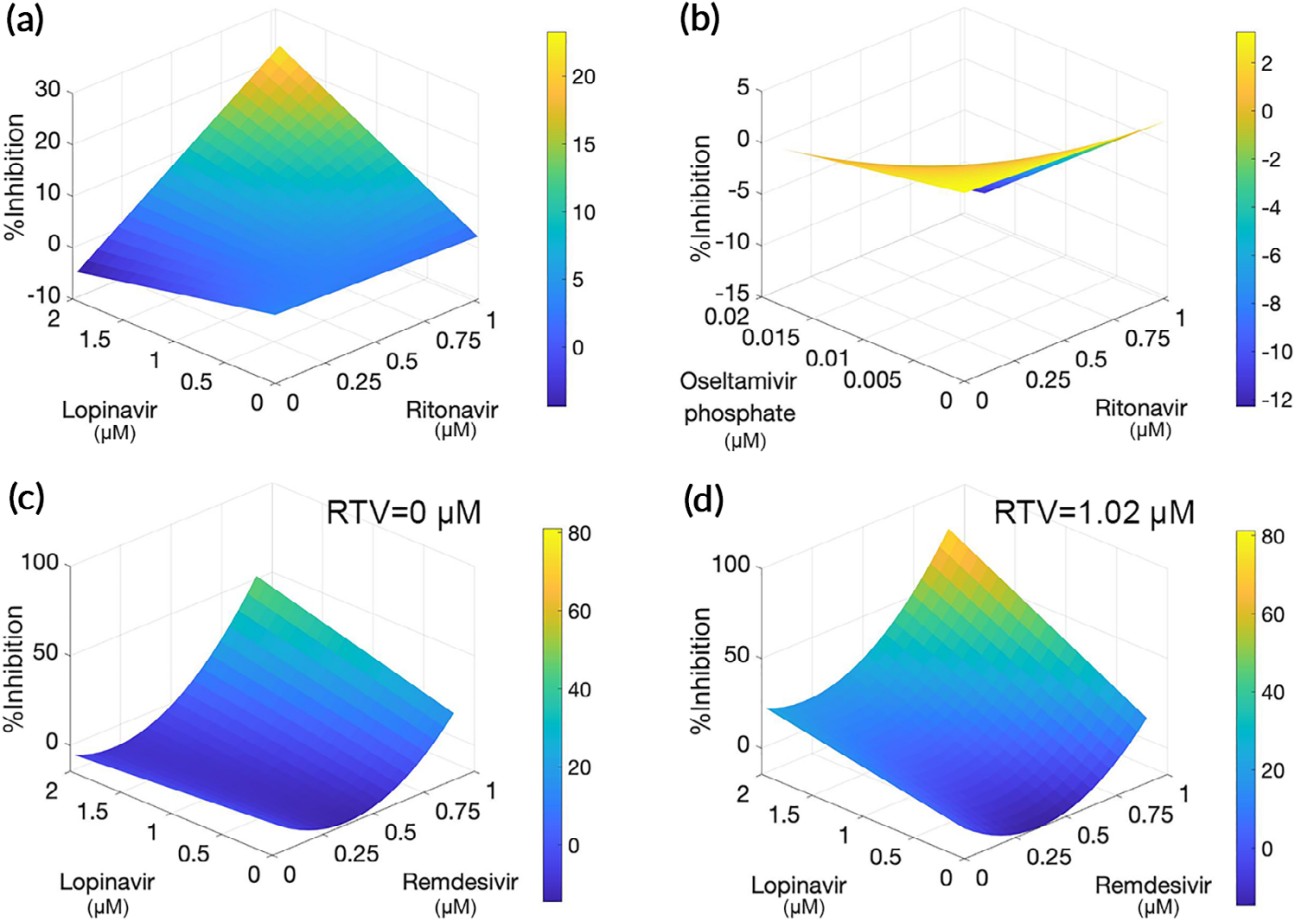
Antiviral drug interactions determined from the IDentif.AI analysis of inhibition in vitro experiments at orthogonal array composite design (OACD) concentration Levels 1 and 2. (a) IDentif.AI determined synergistic interaction between lopinavir and ritonavir (RTV). (b) IDentif.AI determined an antagonistic interaction between RTV and oseltamivir phosphate. (c,d) Synergistic interaction between remdesivir and lopinavir (c) was boosted by the presence of RTV (d)

Drug–drug interactions were investigated with an additional IDentif.AI interaction reanalysis of the OACD experimental data. %Inhibition IDentif.AI response surface plot mirrored well‐documented and experimentally confirmed synergy between RTV and LPV (Figure 3(a)). In contrast, IDentif.AI identified an antagonistic interaction between RTV and OSV‐P (Figure 3(b)), a combination that is currently being investigated in clinical trials (NCT04303299). It is important to note that combining RDV with LPV only at clinically relevant concentrations, which to our knowledge has not been explored clinically as a registered trial, doubled their individual viral CPE inhibition when added together. Accordingly, the corresponding %Inhibition IDentif.AI response surface plot identified a previously unknown synergistic interaction between RDV and LPV (Figure 3(c)). Further confirming IDentif.AI rankings and validation experiments, it is important to note that the RDV/RTV interaction was not significant, but when given in three‐drug combination, RTV boosted the RDV/LPV interaction almost two times (Figure 3(d)). These results further highlight the ability of IDentif.AI to leverage unexpected drug–dose interactions to identify optimal drug combinations at clinically relevant concentrations from a massive drug–dose search space.

## 3 DISCUSSION

This study harnessed the IDentif.AI platform, which paired an AI‐based digital medicine approach with experimental assays and AI‐based optimization on an in vitro infection model to interrogate a 12 drug–dose parameter space at clinically relevant concentrations against the SARS‐CoV‐2 live virus to develop actionable and optimized combination therapy regimens. IDentif.AI addresses several important factors when designing multidrug regimens that are best suited for clinical translation from in vitro validation, especially under urgent scenarios like COVID‐19. Importantly, IDentif.AI considers the critical need for simultaneous reconciliation of drug composition at clinically relevant concentrations and dosing within combination therapy design. MOA‐based drug selection alone followed by dose finding, while an established method of combination therapy design, presents substantial barriers to the optimization process since drug dosing also plays a role in determining which drugs belong in an ideal combination. In lieu of validating a small number of MOA‐based potential drug combinations for efficacy which is commonly observed in traditional workflows, IDentif.AI takes an MOA‐agnostic approach to experimentally test and analyze crowdsourced therapeutic responses to the live virus following expansive drug–dose exposure to both outline the drug–dose space and define resulting drug–dose compositions of the optimal regimens.^52^ With these data, IDentif.AI is able to leverage on unexpected dose‐dependent drug interactions to mediate improved treatment outcomes over MOA‐based drug selection followed by dose finding.

Another critical aspect of IDentif.AI is that the in vitro drug dosing parameter space interrogated in this study is a departure from traditional drug screening approaches. In traditional drug screening, compounds that do not elicit at least a low micromolar EC_50_ treatment response during drug dose–response evaluations are typically removed from further consideration, thereby markedly reducing the number of candidate therapies and possible drug combinations. The removal of these drug candidates is a key driver of suboptimal treatment responses as it ignores a broad spectrum of potential combinations that can be assessed. Lack of monotherapy efficacy does not preclude the use of these drug candidates from IDentif.AI's combinatorial search space. Instead, IDentif.AI's approach allows for continued evaluation of these drugs to determine if they are vital toward driving previously unknown drug interactions that optimize combinatorial treatment outcomes at clinically relevant concentrations. In addition to being observed in this study, this phenomenon has also been observed with our prior clinical studies in chronic infectious diseases and blood and solid cancers, among other indications.^7^, ^10^, ^11^, ^53^

The outcome of applying IDentif.AI toward combating SARS‐CoV‐2 infection is an extensive list of combinations at clinically relevant concentrations ranked by efficacy and/or safety that can be queried by a clinician based on clinically actionable criteria. These include, but are not limited to: highest ranked two‐, three‐, and four‐drug combinations by efficacy; highest ranked combinations that do not contain certain drugs due to supply shortages; highest ranked combinations that do not contain certain drugs or contain lower dosages of certain drugs due to patient comorbidities; and highest ranked combination comprised of only approved therapies, among others (Table 3). In the context of optimized regimen design, which assesses regimen performance from the entire landscape of possible drug/dose parameters, IDentif.AI‐enabled comparative evaluation of the relative efficacy of a broad spectrum of optimized regimens and clinically investigated regimens also independently confirmed the reported outcomes of clinical trials. This provides additional support for the potential application of IDentif.AI as a clinical decision support platform.

For example, the relatively low efficacy exhibited by HCQ alone (3.9% inhibition) or by HCQ and AZT (3% inhibition) in this study aligned with recent reporting of clinical outcomes for this drug given in mono‐ and combinatory therapy, currently demonstrating no evidence of clinical benefit on antiviral efficacy (qualitative PCR assay for SARS‐CoV‐2), time to intubation or death, and 28‐day mortality rate.^50^, ^51^ The relatively low efficacy (3.9 and 5.2% inhibition) of RTV and LPV combination when assessed by IDentif.AI at two different dosing ratios also aligned with recently reported outcomes showing no benefit over SOC.^1^ IDentif.AI also revealed a relatively low efficacy of FPV monotherapy (4.2% inhibition) and in various combinations. This was consistent with clinical findings of FPV being potentially clinically effective, such as shorter viral clearance time and clinical improvement, when administered with interferon‐alpha, not included within our drug library, and as a monotherapy administered to moderate, severe, and critical COVID‐19 patients. Of note, RDV alone resulted in the highest relative efficacy for monotherapy (15.5%) in this study. To date, compassionate use of RDV resulted in clinical improvement of 68% of the patients, and RDV administration in the SIMPLE‐severe trial resulted in a statistically significant improvement in median time to recovery from 15 to 11 days.^4^

Recent results from the RECOVERY trial recruiting moderate, severe, and critical COVID‐19 patients found that DEX reduced 28‐day mortality among COVID‐19 patients who receiving invasive ventilation (DEX: 29% vs. SOC: 40.7%) or supplemental oxygen (DEX: 21% vs. SOC: 25%) at the time of randomization, but not among patients not requiring respiratory support (DEX: 21.5% vs. SOC: 25%) (NCT04381936). DEX treatment within our validation set had low effects in monotherapy (4.9%) with comparable CPE inhibition to HCQ (3.9%) and FPV (4.2%) monotherapies and RTV/LPV (3.9%) combination. This discrepancy is likely because DEX's efficacy demonstrated in the RECOVERY trial is predominantly attributed to its suppression of immunopathology and not its antiviral properties.^54^ As such, it does not directly relate to the viral CPE inhibition measured in our experiments. Coadministration of DEX with RDV/RTV/LPV did not lower the combinations antiviral properties (CPE inhibition: 101.8 vs. 115.1% after addition of DEX), but lowered its cytotoxicity (Vero E6 cytotoxicity: −14.3 to −30.5%; AC16 cytotoxicity: 5.0 to −3.7%; THLE‐2 cytotoxicity: 23.4–11.4%, after an addition of DEX). These results potentially suggest that the DEX treatment is not a contraindication for RDV/RTV/LPV and the beneficial effects in four‐drug combination warrant further exploration. More comparisons between IDentif.AI and reported clinical trial results will be possible with well stratified COVID‐19 severity and unified outcome reporting and definitions of clinical improvement, which is currently being addressed by the international scientific community with the development of the WHO core outcome set for COVID‐19 clinical trials.^55^

The substantial difference in efficacy observed between suboptimal and optimal regimens highlights the importance of leveraging platforms such as IDentif.AI to systematically design combination therapies. This capability, along with the potentially predictive capacity of IDentif.AI for clinical trial outcomes could provide clinicians with an expanded arsenal of evidence‐based candidate treatments and important insights into which potential treatments to further evaluate or potentially avoid under time‐sensitive circumstances.

It is important to note that the results reported here are derived from primarily an in vitro study that focuses on SARS‐CoV‐2 CPEs. The relationship with other measures of viral burden can differ. Further clinical validation of the outlined combinations in randomized controlled trials will be needed. It should also be noted that, while RDV did not mediate a significant clinical benefit in severe COVID‐19 patients, its efficacy in patients with varying disease burden severities should be evaluated further. Furthermore, the mixed reported clinical outcomes support the need for improved regimen design of RDV‐based treatment. In the event of downstream clinical validation of IDentif.AI‐designed combinations, the drug dosage ratios within the combination may vary from those pinpointed by IDentif.AI. In addition, it is possible that the optimal drug combinations may vary between patients due to their severity of infection, comorbidities, and other factors. It is for these reasons that potential downstream trials may be effective at determining the potential clinical benefit of the IDentif.AI‐designed combinations if the enrolled patients are stratified by these aforementioned clinical parameters. The 12‐drug search set used in this study did not include every therapeutic option currently under clinical investigation. Additional studies, including other repurposed compounds, may yield additional highly ranked and effective combination regimens. Also, as IDentif.AI can be applied to novel small molecules, antibody therapies, and other classes of interventions, their inclusion into the drug pool would add further insight into other potentially actionable regimens. Furthermore, given the rapid mutagenicity of RNA viruses like SARS‐CoV‐2, future studies with different drug candidates and different SARS‐CoV‐2 strains may yield different combinations. However, the efficiency and deterministic nature of IDentif.AI allows it to, based on prospective experimental data, derive a ranked list of optimal regimens from a given set of drug candidates against a defined in vitro infectious disease model within 2 weeks. This further supports its potential application as a clinical decision support platform for the optimized design of combination therapy regimens against multiple SARS‐CoV‐2 strains as well as future unknown pathogens that will again require rapid mobilization and clinical guidance for effective treatment options.

## 4 MATERIALS AND METHODS

### 4.1 Design of drug combinations

Drug combinations for 12 drugs at three concentration levels (0, 1, 2) were generated using an OACD as described by Xu et al.^23^ The OACD combines resolution IV two‐level factorial design and a three‐level orthogonal array to provide the least number of combinations required for factor screening of each independent drug's linear, bilinear (drug–drug interaction), and quadratic effects.^11^, ^13^, ^23^ The resolution IV OACD used for this study had 100 combinations: 36 combinations based on the orthogonal array combined with 64 combinations based on the factorial design (Table S1, Supporting Information).

### 4.2 SARS‐CoV‐2 virus

All experiments involving live virus were performed in a biosafety level‐3 (BSL‐3) laboratory. SARS‐CoV‐2 was isolated from a nasopharyngeal swab of a patient in Singapore with ethics approval and consent as described in Reference 12, approved under Domain Specific Review Board study number 2012/00917, additional OSHE/iORC protocol 2020‐00494, and propagated using Vero E6 C1008 cells in minimum Eagle's medium (MEM; Gibco) supplemented with 2% heat‐inactivated fetal bovine serum (HI‐FBS; Gibco). Virus stock was maintained at −80°C. Virus titer was determined by a standard TCID_50_ endpoint dilution assay using Viral ToxGlo Assay (Promega). Briefly, the reagent was added into each well and incubated for 10 min at room temperature prior to measurement of luminescence readout using microplate reader (Tecan).

### 4.3 Cell cultures

African green monkey kidney Vero E6 cells (C1008) were plated at 2 × 10^4^ cells/well density in opaque (white) tissue culture 96‐well plates (Greiner Bio‐One) at the same time as the addition of the drug treatments and virus treatments and cultured in MEM (Gibco) supplemented with 2% HI‐FBS. Human liver epithelial THLE‐2 cells (CRL2706, ATCC) were plated at 3 × 10^3^ cells/well density in 96‐well plates coated with bronchial epithelial cell growth (BEBM) complete medium with fibronectin (0.01 mg/ml; Biological Industries), bovine collagen Type I (0.03 mg/ml; Stem Cell Technologies) and bovine serum albumin (0.01 mg/ml; Sigma‐Aldrich). The BEBM complete medium consisted of BEGM Bullet Kit (Lonza) excluding gentamicin/amphotericin and epinephrine but additionally supplemented with EGF (5 ng/ml), phosphoethanolamine (70 ng/ml), and 10% FBS (Biowest). Human cardiomyocyte AC16 cells (SCC‐09, Millipore) were plated at 2 × 10^3^ cells/well density in 96‐well uncoated plates and cultured in complete AC16 medium—DMEM/F12 (Life Technologies) supplemented with l‐glutamine (2 mm; Life Technologies), 12.5% FBS (Biowest), and 1% penicillin–streptomycin (Life Technologies). All cell cultures were incubated in a humidified atmosphere, at 37°C with 5% CO_2_ atmosphere.

### 4.4 Drugs

RDV (MedChem Express, HY‐104077), FPV (MedChem Express, HY‐14768), (RTV (Selleck Chemicals, S1185), LPV (Selleck Chemicals, cat. no. S1380), RBV (Selleck Chemicals, S2504), AZT (Selleck Chemicals, S1835), LST (Selleck Chemicals, S1359), and DEX (Selleck Chemicals, S1322) were dissolved in DMSO (MP Biomedicals). CQ diphosphate (Selleck Chemicals, S4157), HCQ sulfate (Selleck, S4430), OSV‐P (MedChem Express, HY‐17016), and TEC (Selleck Chemicals, S1399) were dissolved in sterile‐filtered water.

### 4.5 Viral inhibition and cell cytotoxicity of drug monotherapies

All virus infection experiments were performed in a BSL‐3 laboratory. The drugs were diluted in Vero E6 culturing media before dispensing into wells of 96‐well plates. The laboratory staff performing the subsequent experimental work was blinded to the well content arrangement on the plates. The Vero E6 cells (2 × 10^4^ cells/well) and media with and without SARS‐CoV‐2 treatment (100 TCID_50_) were added to the plates containing the drugs and the controls. The drug concentrations ranged between: 1.536 × 10^−3^ μM to 600 μM for FPV, 2.56 × 10^−5^ μM to 10 μM for OSV‐P, 1.28 × 10^−4^ μM to 50 μM for TEC, and 2.56 × 10^−4^ μM to 100 μM for the remaining drugs. Vehicle controls were 0.1% DMSO and media only. After 72 h incubation, cell viability was determined by luminescence‐based ATP activity measurement with Viral ToxGlo (Promega, G8941) as per manufacturer's instructions. The Viral ToxGlo assay quantifies viral‐induced CPEs in host cells by using cellular ATP as a surrogate marker of host cell viability. Upon viral infection, the decrease in cellular ATP detected is proportional to the number of viable host cells in culture, hence, correlating viral CPE with viral burden.

*Z'*‐factor has been calculated to assess the assay quality across all experiments and in each viral experimental set to ensure it can generate reliable information. *Z'*‐factor is a statistical coefficient that incorporates dynamic range and data variability of the positive and negative controls:

(1) $$Z^{\prime }=1-\frac{3\sigma_{c+}+3\sigma_{c-}}{\left|\mu_{c+}-\mu_{c-}\right|}$$

where *σ*_*c*+_ and *μ*_*c*+_ represent the *SD* and mean of the luminescence signal of the positive control (control cells) and *σ*_*c*‐_ and *μ*_*c*‐_ represent the *SD* and mean of the luminescence signal of the negative control (cell + virus control), respectively. 0 < *Z'* < 0.5 represents a “do‐able assay” and 0.5 ≤ *Z'* < 1 represents an “excellent assay.”^56^

Luminescence data were normalized to the average readout from the vehicle control cells on the same plates. Cytotoxicity and viral CPE inhibition were calculated as follows^57^, ^58^:

(2) $$\%\text{Cytotoxicity},T=\frac{\mu_{c+}-E_{+}}{\mu_{c+}}\times 100$$

(3) $$\%\text{Inhibition},I=\frac{E_{-}-\mu_{c-}}{\mu_{c+}-\mu_{c-}}\times 100$$

where *μ*_*c*+_ and *μ*_*c‐*_ represent the mean of the luminescence signal of the positive control (control cells) and negative control (cells + virus control) and *E*_+_ and *E*_*−*_ represent the luminescence signal of each experimental replicate without virus (cells + drugs) and with virus (cells + drugs + virus), respectively. To mitigate the confounding effect of the high cytotoxicity on the viral CPE inhibition calculations, the inhibition values corresponding to drug concentrations resulting in cytotoxicity above 25% were excluded from the analysis. GraphPad Prism 8.2 software (GraphPad Software) was used to plot dose–response curves and to derive efficacy and cytotoxicity concentrations (respectively) at 10, 20, and 50% absolute levels.

### 4.6 Viral inhibition and cell cytotoxicity of drug combinations

All virus infection experiments were performed in a BSL‐3 laboratory. Drug concentrations Levels 1 and 2 for each drug were derived from dose–response curves and clinically relevant values. The drugs in monotherapies and in combinations were dispensed into 96‐well white plates by the automated liquid dispensing system, Mini Janus (PerkinElmer). The laboratory staff performing the subsequent experimental work was blinded to the well content arrangement on the plates. The Vero E6 cells (2 × 10^4^ cells/well) and media with and without SARS‐CoV‐2 treatment (100 TCID_50_) were added to the plates containing the drugs and the controls. The drug combinations and concentrations were prepared according to the OACD table. Vehicle controls were 0.006% DMSO and cells with media only. After 72 h incubation, cell viability was determined by luminescence‐based ATP activity measurement with Viral ToxGlo (Promega, G8941) as per manufacturer's instructions.

Drug cytotoxicity was additionally measured in THLE‐2 human liver and AC16 human cardiomyocyte cell lines. THLE‐2 and AC16 cells were cultured for 24 h prior to treatment with the same drug combinations as those used on Vero E6 cells. After 72 h incubation, THLE‐2 and AC16 cell viability were determined with luminescence‐based ATP activity measurement with CellTiter‐GLO (Promega, G7570) as per manufacturer's instructions.

For validation experiments, selected drug combinations from the IDentif.AI analysis were tested on VeroE6, THLE‐2, and AC16 cells using the same methods as described above. IDentif.AI top ranked combinations, top combinations with and without RDV; combinations exploring drugs and doses interaction space in RDV/RTV/LPV; treatments corresponding to combinatory and monotherapies under current clinical investigation; and RDV/RTV/LPV interaction space with concomitant medications were all interrogated in validation experiments. The %Inhibition confirmed the IDentif.AI‐derived ranking, similar trends were observed in the %Cytotoxicity in THLE‐2 and AC16 cells (Figure 2 and Table 3). No effect of the maximum vehicle concentration (0.006% DMSO) was detected on viral inhibition or on cell cytotoxicity (Wilcoxon rank‐sum test, *N* = 3, *p* > 0.05).

### 4.7 IDentif.AI analysis

IDentif.AI, a dynamic optimization AI‐based platform, identifies the drug–dose parameter space by harnessing the quadratic relationship between biological responses to external perturbations, such as drug/dose inputs.^59^ IDentif.AI analysis of the drug–dose parameter space identifies drug–drug interactions and ranks optimal drug‐dosage combinations. This study aimed to use IDentif.AI to determine effective optimal drug‐dosage combinations from a diverse set of 12 drugs currently being explored in clinical trials to combat the COVID‐19 disease. The concentration levels of the 12 drugs for the in vitro IDentif.AI experiments were determined from EC_50_, CC_50_, and C_max_ for corresponding clinically administered dosages. From the in vitro experiment data, IDentif.AI analyses were performed to identify drug combinations from this pool of candidates that were effective against the SARS‐CoV‐2 virus.

Luminescence data for each well were normalized to the average readout from the DMSO vehicle controls on the same plates. Vero E6, AC16, and THLE‐2 %Cytotoxicity and viral activity %Inhibition (Vero E6) were calculated using the same formulae as for the drug monotherapy analysis. %Inhibition calculations used cell and media only control wells. The resulting %Cytotoxicity and %Inhibition calculations were used as inputs in IDentif.AI analysis.

IDentif.AI analysis correlated drug combinations experimental results into a second‐order quadratic series. Each independent drug combination inhibition and monotherapy inhibition replicate was used in the optimization process. The second‐order quadratic model is as follows:

(4) $$y=\beta_{0}+\beta_{1}x_{1}+\cdots +\beta_{n}x_{n}+\beta_{12}x_{1}x_{2}+\cdots \beta_{\mathrm{mn}}x_{m}x_{n}+\beta_{11}x_{1}^{2}+\cdots +\cdots +\beta_{\mathrm{nn}}x_{n}^{2}$$

where *y* represents the desired biological response output (%Inhibition), *x*_*n*_ is the *n*th drug concentration, *β*_0_ is the intercept term, *β*_*n*_ is the single‐drug coefficient of the *n*th drug, *β*_*mn*_ is the interaction coefficient between the *m*th and *n*th drugs, and *β*_*nn*_ is the second‐order coefficient for the *n*th drug, while *m* ≠ *n*. This second‐order quadratic analysis and parabolic response surface plot analysis were conducted using the built‐in “*stepwiselm*” function in MATLAB R2020a (MathWorks, Inc.). IDentif.AI derived four quadratic series using bidirectional elimination approach with the *p* value from the F‐statistic as the selection criterion for the experimental results: %Inhibition, %Cytotoxicity, %Cytotoxicity AC16, and %Cytotoxicity THLE‐2. Residual‐based outlier analysis was performed for all four IDentif.AI series. Single replicates identified as outliers remained in the data set to account for biological variation. The combinations with all replicates identified as outliers were excluded from the data set and the IDentif.AI analysis was repeated.

IDentif.AI analysis yielded both drug–drug interaction plots and optimized drug combinations. The optimized drug combinations were ranked according to corresponding %Inhibition from the correlated second‐order quadratic series with the %Cytotoxicity of the cell‐lines (Vero E6, AC16, and THLE‐2) serving as qualitative indicators for consideration. The predictive power was also calculated via adjusted *R* ^2^ to establish the robustness of IDentif.AI optimization considering the number of drug and drug–drug interaction terms. Correlation coefficients were derived from the experimental output values and projected output values for the corresponding drug combinations.

### 4.8 Statistical analysis

All experiments were performed in at least triplicate biological repeats. To account for uncertainties propagated in the process of normalization, %Inhibition and %Cytotoxicity are presented as mean ± propagated *SD*, with the propagated *SD* derived from the following equation^60^:

(5) $$\sigma_{T}^{2}={\left(\frac{\partial T}{\partial c_{+}}\right)}^{2}\sigma_{c_{+}}^{2}+{\left(\frac{\partial T}{\partial E_{+}}\right)}^{2}\sigma_{E_{+}}^{2}$$

(6) $$\sigma_{I}^{2}={\left(\frac{\partial I}{\partial E_{-}}\right)}^{2}\sigma_{E_{-}}^{2}+{\left(\frac{\partial I}{\partial c_{-}}\right)}^{2}\sigma_{c-}^{2}+{\left(\frac{\partial I}{\partial c_{+}}\right)}^{2}\sigma_{c+}^{2}$$

where and *σ*_*T*_ and *σ*_*I*_ represent the propagated *SD* for the mean value of %Cytotoxicity and %Inhibition, and *σ*_*c*+_, *σ*_*c*−_ and *σ*_*E*+,_ *σ*_*E*−_ represent the *SD* of the luminescence signal of the positive control (control cells), negative control (cells + cells + virus control), and the experimental replicates without virus (cells + drugs) and with virus (cells + drugs + virus), respectively. Shapiro–Wilk normality test was used to determine if samples were from normally distributed populations. Variance equality was tested with Bartlett's test. The Kruskal–Wallis test by ranks was used for multiple comparisons, followed by Dunn's post hoc test for pairwise comparisons. Student's two‐tailed *t* test and Wilcoxon rank sum test were used for comparing individual samples from normally and non‐normally distributed populations, respectively. Bonferroni post hoc correction was applied to account for multiple comparisons. Statistical analyses for coefficient estimation in the IDentif.AI analyses were performed using sum of squares *F*‐test. Alongside the *p*‐values, the results were interpreted in the light of logic, background knowledge and the specifics of the experimental design.^47^

### 4.9 Code availability

IDentif.AI analyses were conducted using the built‐in “stepwiselm” function in MATLAB R2020a (MathWorks, Inc.), with example, MATLAB code provided in Supplementary Software as published previously.^11^, ^53^

## 5 CONCLUSIONS

Following the emergence of SARS‐CoV‐2, a global effort to clinically assess a broad spectrum of repurposed and novel compounds was initiated. In order to fully optimize the development of a treatment regimen against SARS‐CoV‐2 or any future epidemic/pandemic, it is important to move beyond traditional drug selection approaches, since mechanism‐of‐action‐based drug selection alone will unlikely yield sufficient efficacy for broadly favorable clinical outcomes. This is because globally optimized combination design will rely on simultaneously optimal drug and dose identification, which is a major challenge for traditional drug screening and repurposing approaches due to an insurmountably large drug–dose parameter space. This work has addressed this challenge using IDentif.AI, an AI‐based digital drug development platform that rapidly crowdsourced the patient‐derived live virus to experimentally pinpoint and validate ranked combinations within 2 weeks. Unpredictable drug interactions were harnessed by IDentif.AI to pinpoint unforeseen, top‐ranked combinations, and the IDentif.AI rankings independently aligned with broadly reported clinical trial outcomes without requiring data from these studies. Therefore, IDentif.AI can be potentially deployed as a first line of defense to rationally pinpoint optimal drug–dose combination therapy regimens for rapid clinical validation while also potentially deterring the assessment of regimens that are unlikely to yield suitable clinical outcomes. Collectively, these capabilities may serve as a foundation for global accessibility to clinically actionable and optimized therapeutic responses to current and future pandemics.

## AUTHOR CONTRIBUTIONS

**Agata Blasiak:** Data curation; formal analysis; investigation; methodology; project administration; supervision; validation; writing‐original draft; writing‐review and editing. **Jhin Jieh Lim:** Data curation; formal analysis; investigation; methodology; validation; visualization; writing‐original draft; writing‐review and editing. **Shirley Gek Kheng Seah:** Data curation; formal analysis; investigation; methodology; supervision; validation; writing‐review and editing. **Theodore Kee:** Data curation; formal analysis; investigation; methodology; software; validation; visualization; writing‐original draft; writing‐review and editing. **Alexandria Remus:** Data curation; formal analysis; investigation; methodology; validation; visualization; writing‐original draft; writing‐review and editing. **De Hoe Chye:** Formal analysis; investigation; validation; writing‐original draft; writing‐review and editing. **Pui San Wong:** Formal analysis; investigation; validation; writing‐original draft; writing‐review and editing. **Lissa Hooi:** Formal analysis; investigation; validation; writing‐original draft; writing‐review and editing. **Anh Truong:** Formal analysis; investigation; software; validation; writing‐original draft; writing‐review and editing. **Nguyen Le:** Formal analysis; investigation; validation; writing‐original draft; writing‐review and editing. **Conrad En Zuo Chan:** Formal analysis; investigation; validation; writing‐original draft; writing‐review and editing. **Rishi Desai:** Investigation; validation; writing‐original draft; writing‐review and editing. **Xianting Ding:** Formal analysis; funding acquisition; investigation; methodology; project administration; writing‐original draft; writing‐review and editing. **Brendon Hanson:** Conceptualization; formal analysis; funding acquisition; investigation; methodology; project administration; supervision; writing‐original draft; writing‐review and editing. **Edward Chow:** Formal analysis; funding acquisition; investigation; methodology; project administration; supervision; writing‐original draft; writing‐review and editing. **Dean Ho:** Conceptualization; formal analysis; funding acquisition; investigation; methodology; project administration; resources; supervision; validation; writing‐original draft; writing‐review and editing.

## CONFLICT OF INTEREST

A. B., T. K., L. H., X. D., E. K‐.H. C., and D. H. are co‐inventors or previously filed pending patents on artificial intelligence‐based therapy development. T. K. E. K.‐H. C., and D. H. are shareholders of KYAN Therapeutics, which has licensed intellectual property pertaining to AI‐based drug development. No intellectual property rights from this reported work are being pursued.

### PEER REVIEW

The peer review history for this article is available at https://publons.com/publon/10.1002/btm2.10196.

## Supporting information


**Appendix S1:** Supplementary Information

## Data Availability

The data supporting the findings of this study are available from the corresponding author upon reasonable request.

## References

[1] Cao B , Wang Y , Wen D , et al. A trial of lopinavir‐ritonavir in adults hospitalized with severe Covid‐19. N Engl J Med. 2020;382(19):1787‐1799. 10.1056/NEJMoa2001282. 32187464 PMC7121492

[2] Gautret P , Lagier JC , Parola P , et al. Hydroxychloroquine and azithromycin as a treatment of COVID‐19: results of an open‐label non‐randomized clinical trial. Int J Antimicrob Agents. 2020;56(1):105949 10.1016/j.ijantimicag.2020.105949. 32205204 PMC7102549

[3] Grein J , Ohmagari N , Shin D , et al. Compassionate use of remdesivir for patients with severe Covid‐19. N Engl J Med. 2020;382(24):2327‐2336. 10.1056/NEJMoa2007016. 32275812 PMC7169476

[4] NIH . NIH clinical trial shows Remdesivir accelerates recovery from advanced COVID‐19. NIH. https://www.nih.gov/news-events/news-releases/nih-clinical-trial-shows-remdesivir-accelerates-recovery-advanced-covid-19. Accessed 29 Apr, 2020

[5] Cai Q , Yang M , Liu D , et al. Experimental treatment with favipiravir for COVID‐19: an open‐label control study. Engineering. 2020 (Epub ahead of print). 10.1016/j.eng.2020.03.007. PMC7185795 32346491

[6] Wang M , Cao R , Zhang L , et al. Remdesivir and chloroquine effectively inhibit the recently emerged novel coronavirus (2019‐nCoV) in vitro. Cell Res. 2020;30(3):269‐271. 10.1038/s41422-020-0282-0. 32020029 PMC7054408

[7] Abdulla A , Wang B , Qian F , et al. Project IDentif.AI: harnessing artificial intelligence to rapidly optimize combination therapy development for infectious disease intervention. Adv Ther. 2020;3(7):2000034 10.1002/adtp.2020000348. PMC7235487 32838027

[8] Ho D . Artificial intelligence in cancer therapy. Science. 2020;367(6481):982‐983. 10.1126/science.aaz3023. 32108102

[9] Zimmer A , Katzir I , Dekel E , Mayo AE , Alon U . Prediction of multidimensional drug dose responses based on measurements of drug pairs. Proc Natl Acad Sci U S A. 2016;113(37):10442‐10447. 10.1073/pnas.1606301113. 27562164 PMC5027409

[10] Tekin E , Beppler C , White C , Mao Z , Savage VM , Yeh PJ . Enhanced identification of synergistic and antagonistic emergent interactions among three or more drugs. J R Soc Interface. 2016;13(119):20160332 10.1098/rsif.2016.0332. 27278366 PMC4938094

[11] Rashid M , Toh TB , Hooi L , et al. Optimizing drug combinations against multiple myeloma using a quadratic phenotypic optimization platform (QPOP). Sci Transl Med. 2018;10(453):eaan0941 10.1126/scitranslmed.aan0941. 30089632

[12] Young BE , Ong SWX , Kalimuddin S , et al. Epidemiologic features and clinical course of patients infected with SARS‐CoV‐2 in Singapore. Jama. 2020;323(15):1488‐1494. 10.1001/jama.2020.3204. 32125362 PMC7054855

[13] Lim JJ , Goh J , Rashid MBMA , EK‐H C . Maximizing efficiency of artificial intelligence‐driven drug combination optimization through minimal resolution experimental design. Adv Ther. 2020;3(4):1900122 10.1002/adtp.201900122.

[14] Pantuck AJ , Lee D‐K , Kee T , et al. Modulating BET Bromodomain inhibitor ZEN‐3694 and enzalutamide combination dosing in a metastatic prostate cancer patient using CURATE.AI, an artificial intelligence platform. Adv Ther. 2018;1(6):1800104 10.1002/adtp.201800104.

[15] Zarrinpar A , Lee DK , Silva A , et al. Individualizing liver transplant immunosuppression using a phenotypic personalized medicine platform. Sci Transl Med. 2016;8(333):333ra49 10.1126/scitranslmed.aac5954. 27053773

[16] de Mel S , Rashid MBM , Zhang XY , et al. Application of an ex‐vivo drug sensitivity platform towards achieving complete remission in a refractory T‐cell lymphoma. Blood Cancer J. 2020;10(1):9 10.1038/s41408-020-0276-7. 31988286 PMC6985240

[17] Harrison C . Coronavirus puts drug repurposing on the fast track. Nat Biotechnol. 2020;38(4):379‐381. 10.1038/d41587-020-00003-1. 32205870

[18] Damle B , Vourvahis M , Wang E , Leaney J , Corrigan B . Clinical pharmacology perspectives on the antiviral activity of azithromycin and use in COVID‐19. Clin Pharmacol Ther. 2020;108(2):201‐211. 10.1002/cpt.1857. 32302411 PMC7262099

[19] Sanders JM , Monogue ML , Jodlowski TZ , Cutrell JB . Pharmacologic treatments for coronavirus disease 2019 (COVID‐19): a review. Jama. 2020;323(18):1824‐1836. 10.1001/jama.2020.6019. 32282022

[20] Zhou N , Pan T , Zhang J , et al. Glycopeptide antibiotics potently inhibit cathepsin L in the late endosome/lysosome and block the entry of Ebola virus, middle east respiratory syndrome coronavirus (MERS‐CoV), and severe acute respiratory syndrome coronavirus (SARS‐CoV). J Biol Chem. 2016;291(17):9218‐9232. 10.1074/jbc.M116.716100. 26953343 PMC4861487

[21] NIH . Considerations for certain concomitant medications in patients with COVID‐19. NIH. https://www.covid19treatmentguidelines.nih.gov/concomitant-medications/. Accessed 29th April 2020

[22] Shea KW , Cunha BA . Teicoplanin. Med Clin North Am. 1995;79(4):833‐844. 10.1016/s0025-7125(16)30042-6. 7791426

[23] Xu H , Jaynes J , Ding X . Combining two‐level and three‐level orthogonal arrays for factor screening and response surface exploration. Stat Sin. 2014;24:269‐289. 10.5705/ss.2012.210.

[24] Yao X , Ye F , Zhang M , et al. In vitro antiviral activity and projection of optimized dosing design of hydroxychloroquine for the treatment of severe acute respiratory syndrome coronavirus 2 (SARS‐CoV‐2). Clin Infect Dis. 2020;71(15):732‐739. 10.1093/cid/ciaa237. 32150618 PMC7108130

[25] Zumla A , Chan JF , Azhar EI , Hui DS , Yuen KY . Coronaviruses—drug discovery and therapeutic options. Nat Rev Drug Discov. 2016;15(5):327‐347. 10.1038/nrd.2015.37. 26868298 PMC7097181

[26] Varga Z , Flammer AJ , Steiger P , et al. Endothelial cell infection and endotheliitis in COVID‐19. Lancet. 2020;395(10234):1417‐1418. 10.1016/S0140-6736(20)30937-5. 32325026 PMC7172722

[27] Ackermann M , Verleden SE , Kuehnel M , et al. Pulmonary vascular endothelialitis, thrombosis, and angiogenesis in Covid‐19. N Engl J Med. 2020;383(2):120‐128. 10.1056/NEJMoa2015432. 32437596 PMC7412750

[28] Remdesivir: Summary on Compassionate Use (EMA) 43; 2020.

[29] Avigan: Review Report (PMDA) 172; 2011.

[30] Hsu A , Granneman GR , Bertz RJ . Ritonavir. Clinical pharmacokinetics and interactions with other anti‐HIV agents. Clin Pharmacokinet. 1998;35(4):275‐291. 10.2165/00003088-199835040-00002. 9812178

[31] Norvir: European Public Assessment Report ‐ Scientific Discussion (EMA); 2005.

[32] Norvir: European Public Assessment Report ‐ Product Information (EMA); 2019.

[33] la Porte CJ , Colbers EP , Bertz R , et al. Pharmacokinetics of adjusted‐dose lopinavir‐ritonavir combined with rifampin in healthy volunteers. Antimicrob Agents Chemother. 2004;48(5):1553‐1560. 10.1128/aac.48.5.1553-1560.2004. 15105105 PMC400571

[34] Breilh D , Foucher J , Castera L , et al. Impact of ribavirin plasma level on sustained virological response in patients treated with pegylated interferon and ribavirin for chronic hepatitis C. Aliment Pharmacol Ther. 2009;30(5):487‐494. 10.1111/j.1365-2036.2009.04065.x. 19523176

[35] Pereira D , Daher A , Zanini G , et al. Safety, efficacy and pharmacokinetic evaluations of a new coated chloroquine tablet in a single‐arm open‐label non‐comparative trial in Brazil: a step towards a user‐friendly malaria vivax treatment. Malar J. 2016;15:477 10.1186/s12936-016-1530-0. 27639847 PMC5027105

[36] Plaquenil: Label (FDA); 2017.

[37] Zithromax: Label (FDA); 2013.

[38] Jittamala P , Pukrittayakamee S , Tarning J , et al. Pharmacokinetics of orally administered oseltamivir in healthy obese and nonobese Thai subjects. Antimicrob Agents Chemother. 2014;58(3):1615‐1621. 10.1128/AAC.01786-13. 24366750 PMC3957867

[39] Pai MP , Lodise TP Jr . Oseltamivir and oseltamivir carboxylate pharmacokinetics in obese adults: dose modification for weight is not necessary. Antimicrob Agents Chemother. 2011;55(12):5640‐5645. 10.1128/AAC.00422-11. 21930881 PMC3232799

[40] Tamiflu: Highlights of Prescribing Information (FDA); 2016.

[41] Cozaar: Highlights of Prescribing Information (FDA); 2018.

[42] Ohtawa M , Takayama F , Saitoh K , Yoshinaga T , Nakashima M . Pharmacokinetics and biochemical efficacy after single and multiple oral administration of losartan, an orally active nonpeptide angiotensin II receptor antagonist, in humans. Br J Clin Pharmacol. 1993;35(3):290‐297. 10.1111/j.1365-2125.1993.tb05696.x. 8471405 PMC1381577

[43] Nah SY , Im JH , Yeo JY , et al. Therapeutic drug concentrations of teicoplanin in clinical settings. Infect Chemother. 2014;46(1):35‐41. 10.3947/ic.2014.46.1.35. 24693468 PMC3970309

[44] Hemady: Highlights of Prescribing Information (FDA); 2019.

[45] Sica DA , Gehr TW , Ghosh S . Clinical pharmacokinetics of losartan. Clin Pharmacokinet. 2005;44(8):797‐814. 10.2165/00003088-200544080-00003. 16029066

[46] Dykhuizen RS , Harvey G , Stephenson N , Nathwani D , Gould IM . Protein binding and serum bactericidal activities of vancomycin and teicoplanin. Antimicrob Agents Chemother. 1995;39(8):1842‐1847. 10.1128/aac.39.8.1842. 7486929 PMC162836

[47] It's time to talk about ditching statistical significance. Nature. 2019;567(7748):283 10.1038/d41586-019-00874-8. 30894740

[48] Kuwajima K , Chang K , Furuta A , et al. Synergistic cytoprotection by co‐treatment with dexamethasone and rapamycin against proinflammatory cytokine‐induced alveolar epithelial cell injury. J Intensive Care. 2019;7(1):12 10.1186/s40560-019-0365-5. 30774959 PMC6367811

[49] Mattern J , Büchler MW , Herr I . Cell cycle arrest by glucocorticoids may protect normal tissue and solid tumors from cancer therapy. Cancer Biol Ther. 2007;6(9):1345‐1354. 10.4161/cbt.6.9.4765. 18087223

[50] Chen J , Liu D , Liu L , et al. A pilot study of hydroxychloroquine in treatment of patients with common coronavirus disease‐19 (COVID‐19). J Zhejiang Univ Med Sci. 2020;49(2):215‐219. 10.3785/j.issn.1008-9292.2020.03.03. PMC8800713 32391667

[51] Molina JM , Delaugerre C , le Goff J , et al. No evidence of rapid antiviral clearance or clinical benefit with the combination of hydroxychloroquine and azithromycin in patients with severe COVID‐19 infection. Med Mal Infect. 2020;50(4):384 10.1016/j.medmal.2020.03.006. 32240719 PMC7195369

[52] Ho D . Addressing COVID‐19 drug development with artificial intelligence. Adv Intell Syst. 2020;2(5):2000070 10.1002/aisy.202000070. PMC7235485 32838299

[53] Silva A , Lee BY , Clemens DL , et al. Output‐driven feedback system control platform optimizes combinatorial therapy of tuberculosis using a macrophage cell culture model. Proc Natl Acad Sci U S A. 2016;113(15):E2172‐E2179. 10.1073/pnas.1600812113. 27035987 PMC4839402

[54] The RECOVERY Collaborative Group . Dexamethasone in hospitalized patients with Covid‐19—preliminary report. N Engl J Med. 2020 10.1056/NEJMoa2021436. PMC7383595 32678530

[55] Marshall JC , Murthy S , Diaz J , et al. A minimal common outcome measure set for COVID‐19 clinical research. Lancet Infect Dis. 2020;20(8):e192‐e197. 10.1016/S1473-3099(20)30483-7. 32539990 PMC7292605

[56] Zhang J‐H , Chung TDY , Oldenburg KR . A simple statistical parameter for use in evaluation and validation of high throughput screening assays. J Biomol Screen. 1999;4(2):67‐73. 10.1177/108705719900400206. 10838414

[57] Zhang M , Zhang Y , Wang Y , Lv W , Zhang Y . Automated cell‐based luminescence assay for profiling antiviral compound activity against enteroviruses. Sci Rep. 2019;9(1):6023 10.1038/s41598-019-42160-7. 30988314 PMC6465263

[58] Andrighetti‐Frohner CR , Antonio RV , Creczynski‐Pasa TB , Barardi CR , Simoes CM . Cytotoxicity and potential antiviral evaluation of violacein produced by *Chromobacterium violaceum* . Mem Inst Oswaldo Cruz. 2003;98(6):843‐848. 10.1590/s0074-02762003000600023. 14595466

[59] Al‐Shyoukh I , Yu F , Feng J , et al. Systematic quantitative characterization of cellular responses induced by multiple signals. BMC Syst Biol. 2011;5:88 10.1186/1752-0509-5-88. 21624115 PMC3138445

[60] Farrance I , Frenkel R . Uncertainty of measurement: a review of the rules for calculating uncertainty components through functional relationships. Clin Biochem Rev. 2012;33(2):49‐75. 22896744 PMC3387884

