# Supplementary material for: IDentif.AI: Rapidly optimizing combination therapy design against severe Acute Respiratory Syndrome Coronavirus 2 (SARS‐Cov‐2) with digital drug development
Source: Bioeng Transl Med. 2020 Dec 1;6(1):e10196. doi: 10.1002/btm2.10196 (PMC7823122; doi:10.1002/btm2.10196)
Supplement: Supplementary file 1 — Appendix S1: Supplementary Information [file BTM2-6-e10196-s001.docx]

IDentif.AI: Rapidly Optimizing Combination Therapy Against Severe Acute Respiratory Syndrome Coronavirus 2 (SARS-CoV-2) with Artificial Intelligence

Agata Blasiak^#^, Jhin Jieh Lim^#^, Shirley Gek Kheng Seah^#^, Theodore Kee^#^, Alexandria Remus^#^, De Hoe Chye, Pui San Wong, Lissa Hooi, Anh T.L. Truong, Nguyen Le, Conrad E.Z. Chan, Rishi Desai, Xianting Ding*, Brendon J. Hanson*, Edward Kai-Hua Chow*, and Dean Ho*

Supporting Information


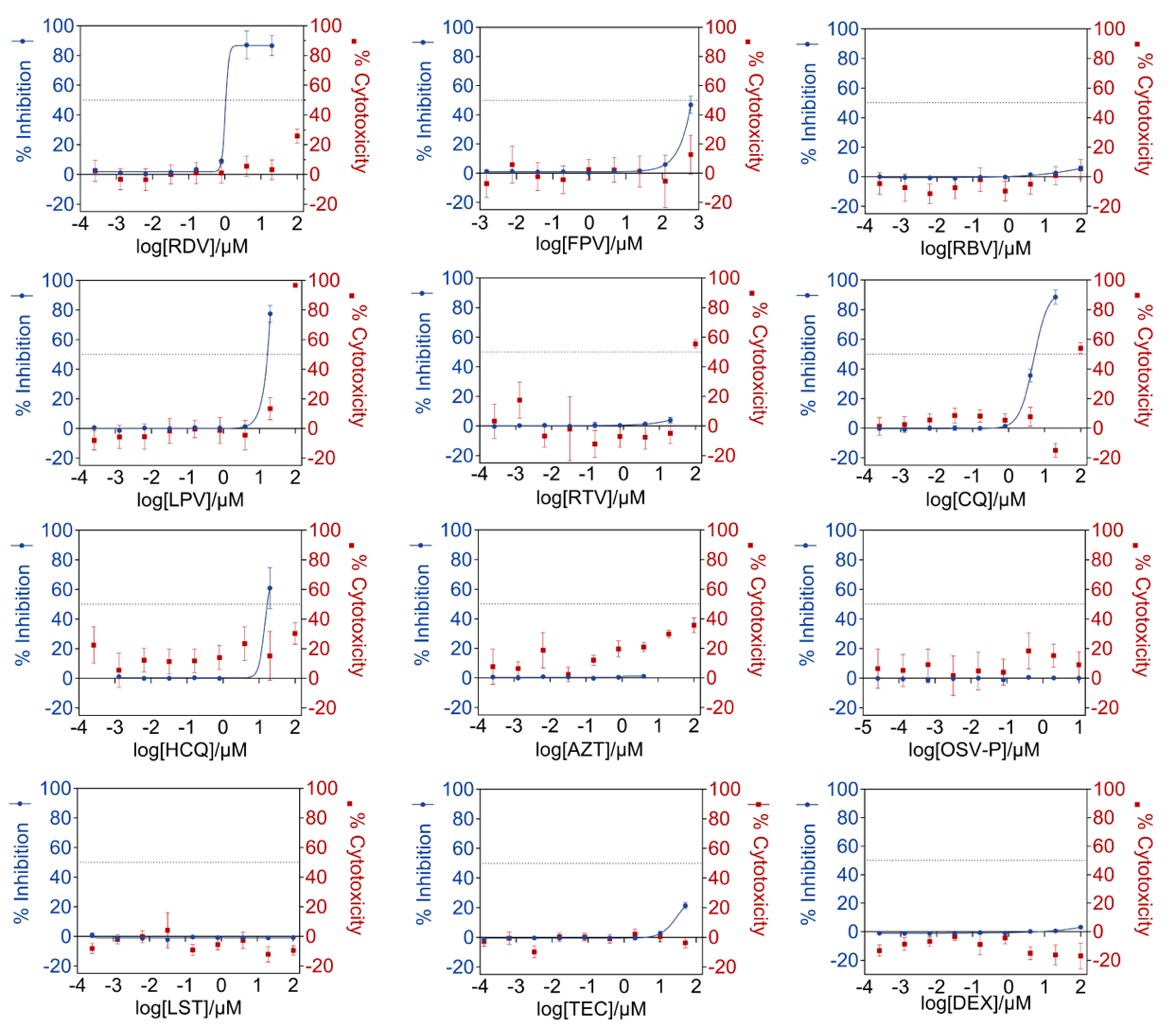


**Figure S1.** Dose-response curves for drugs given in monotherapies. Vero E6 cells were infected with SARS-CoV-2 at 100 TCID_50_ in the treatment with different drug concentrations for 72 h. Viral infection inhibition and cytotoxicity of these drugs to Vero E6 cells were determined by measuring cell viability with luminescence-based ATP activity assay. The left and right *Y*-axis of the graphs represent mean %viral infection inhibition and %cytotoxicity of the drugs, respectively. The experiments were done in triplicates. Each value is represented as mean ± propagated SD. Remdesivir (RDV), favipiravir (FPV), ritonavir (RTV), lopinavir (LPV), ribavirin (RBV), azithromycin (AZT), losartan (LST), dexamethasone (DEX), chloroquine diphosphate (CQ), hydroxychloroquine sulfate (HCQ), oseltamivir phosphate (OSV-P) and teicoplanin (TEC).


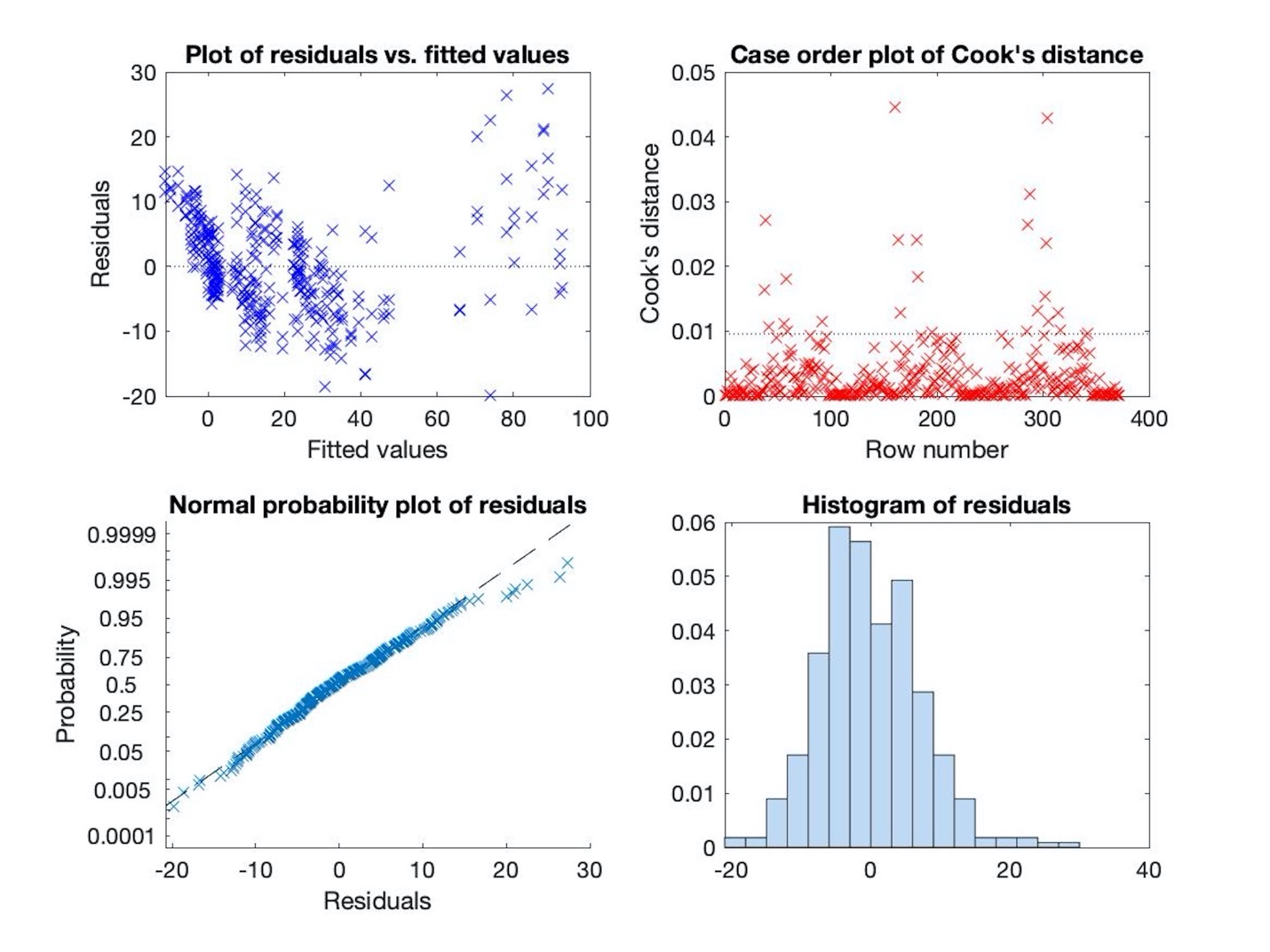


**Figure S2.** Outlier analysis for individual replicates in IDentif.AI %Inhibition analysis. No data points were removed from the subsequent analysis. Row number in Cook’s distance plot corresponds to each combination’s triplicates in an order specified by the OACD table.


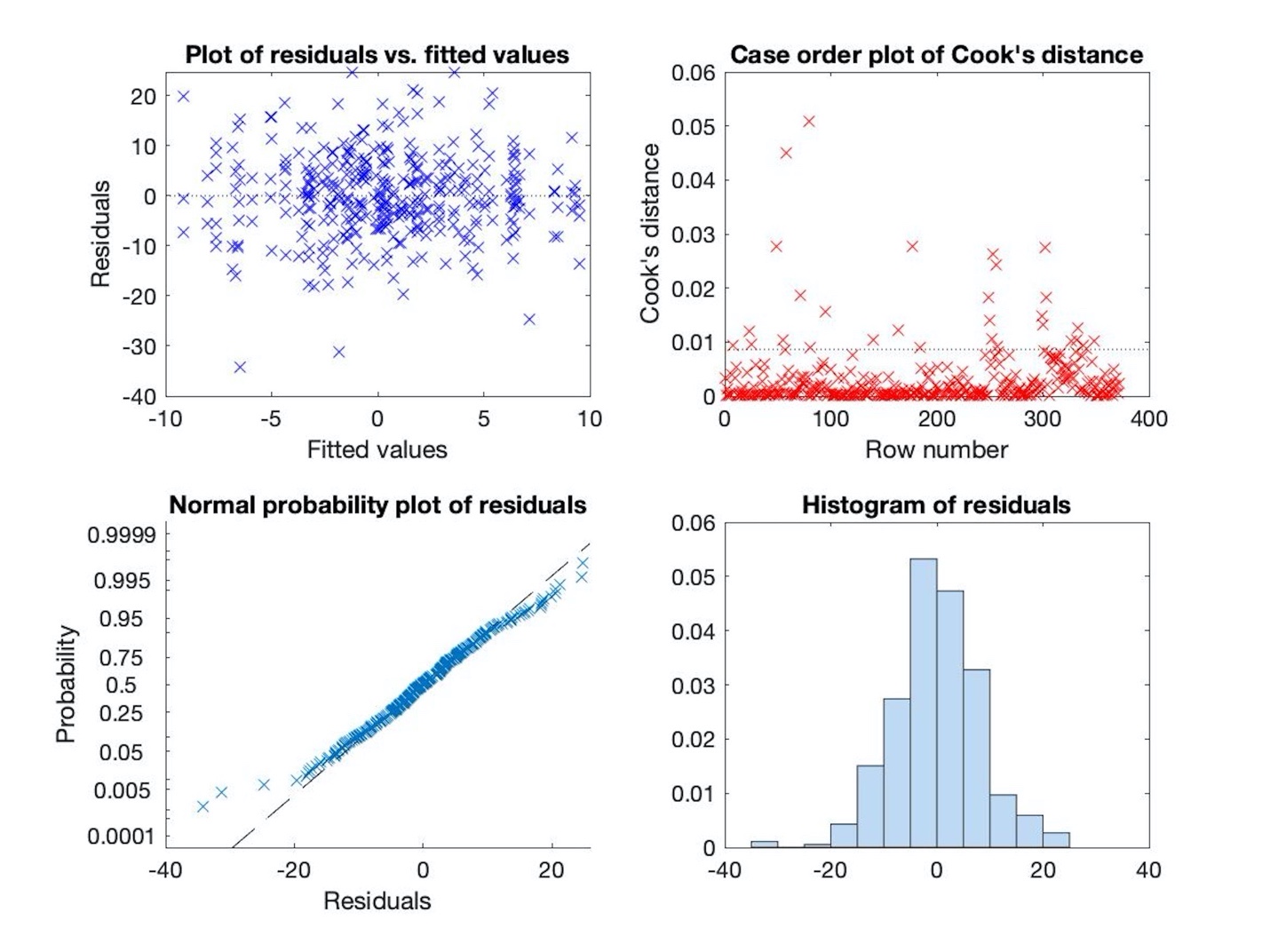


**Figure S3.** Outlier analysis for individual replicates in IDentif.AI Vero E6 %Cytotoxicity analysis. No data points were removed from the subsequent analysis. Row number in Cook’s distance plot corresponds to each combination’s triplicates in an order specified by the OACD table.


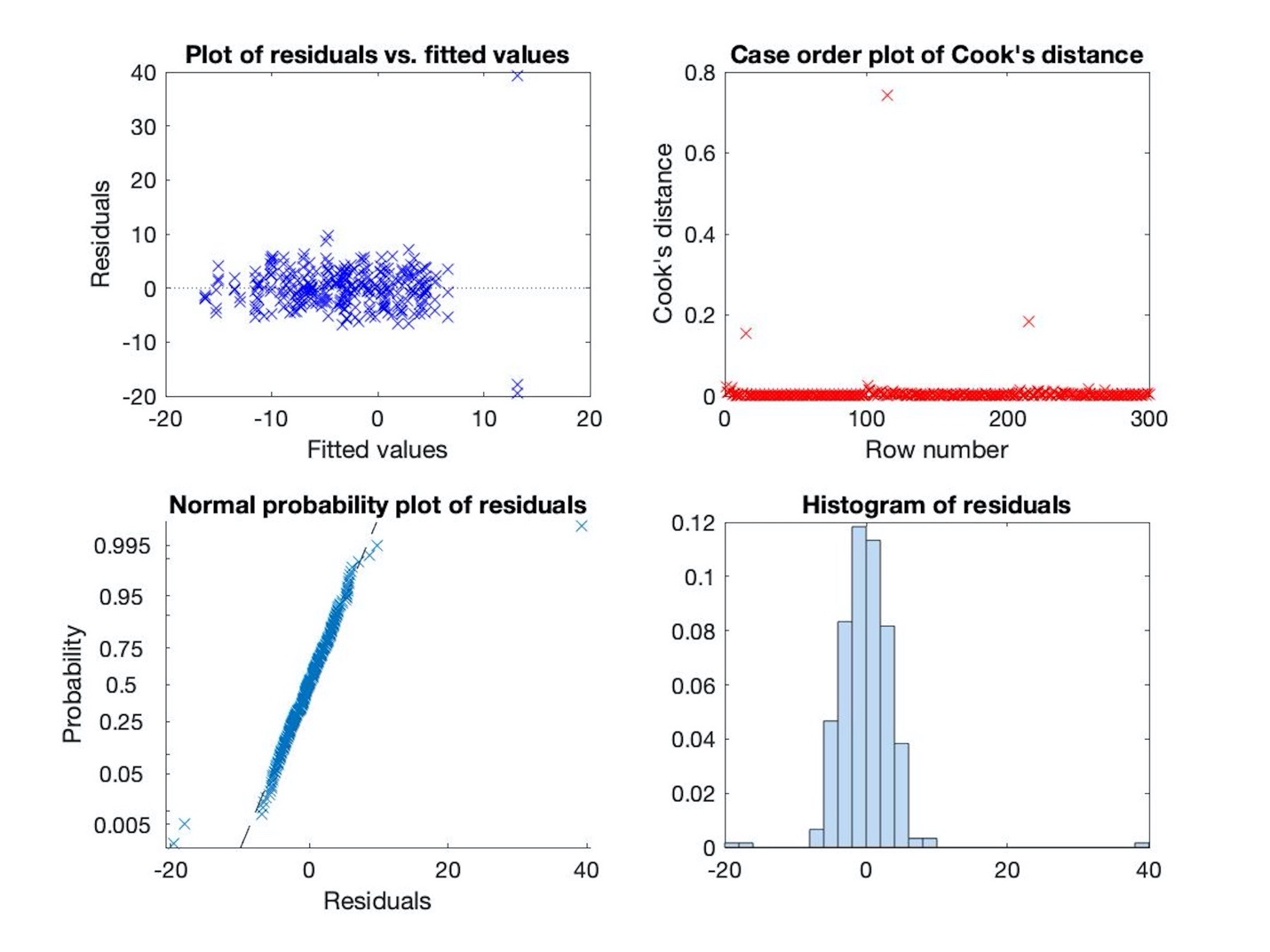


**Figure S4.** Outlier analysis for individual replicates in IDentif.AI AC16 %Cytotoxicity analysis. All three replicates corresponding to combination 15 were identified as outliers and were removed for subsequent analysis. Row number in Cook’s distance plot corresponds to each combination’s triplicates in an order specified by the OACD table.


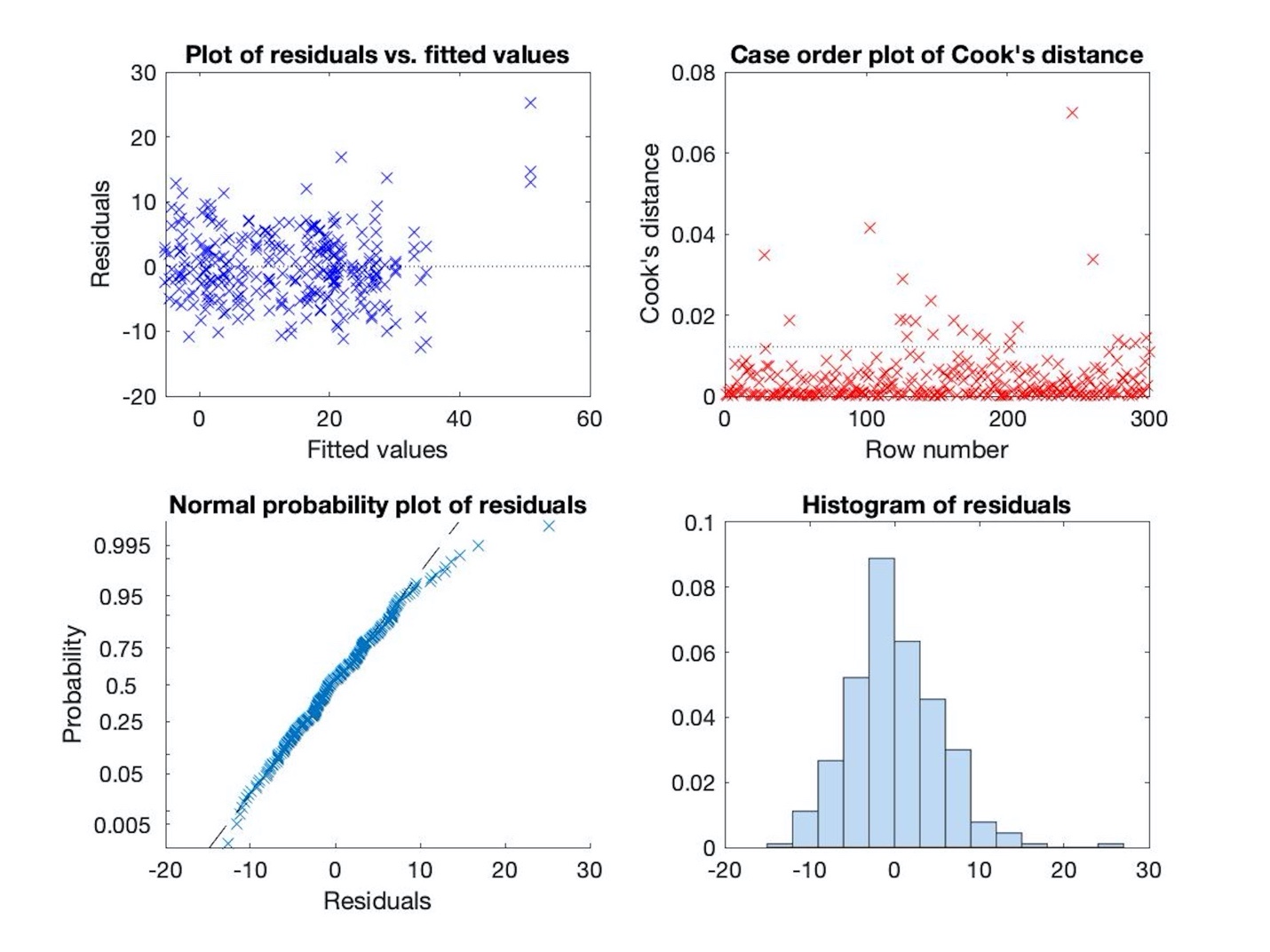


**Figure S5.** Outlier analysis for individual replicates in IDentif.AI THLE-2 %Cytotoxicity analysis. All three replicates corresponding to combination 46 were identified as outliers and were removed for subsequent analysis. Row number in Cook’s distance plot corresponds to each combination’s triplicates in an order specified by the OACD table.


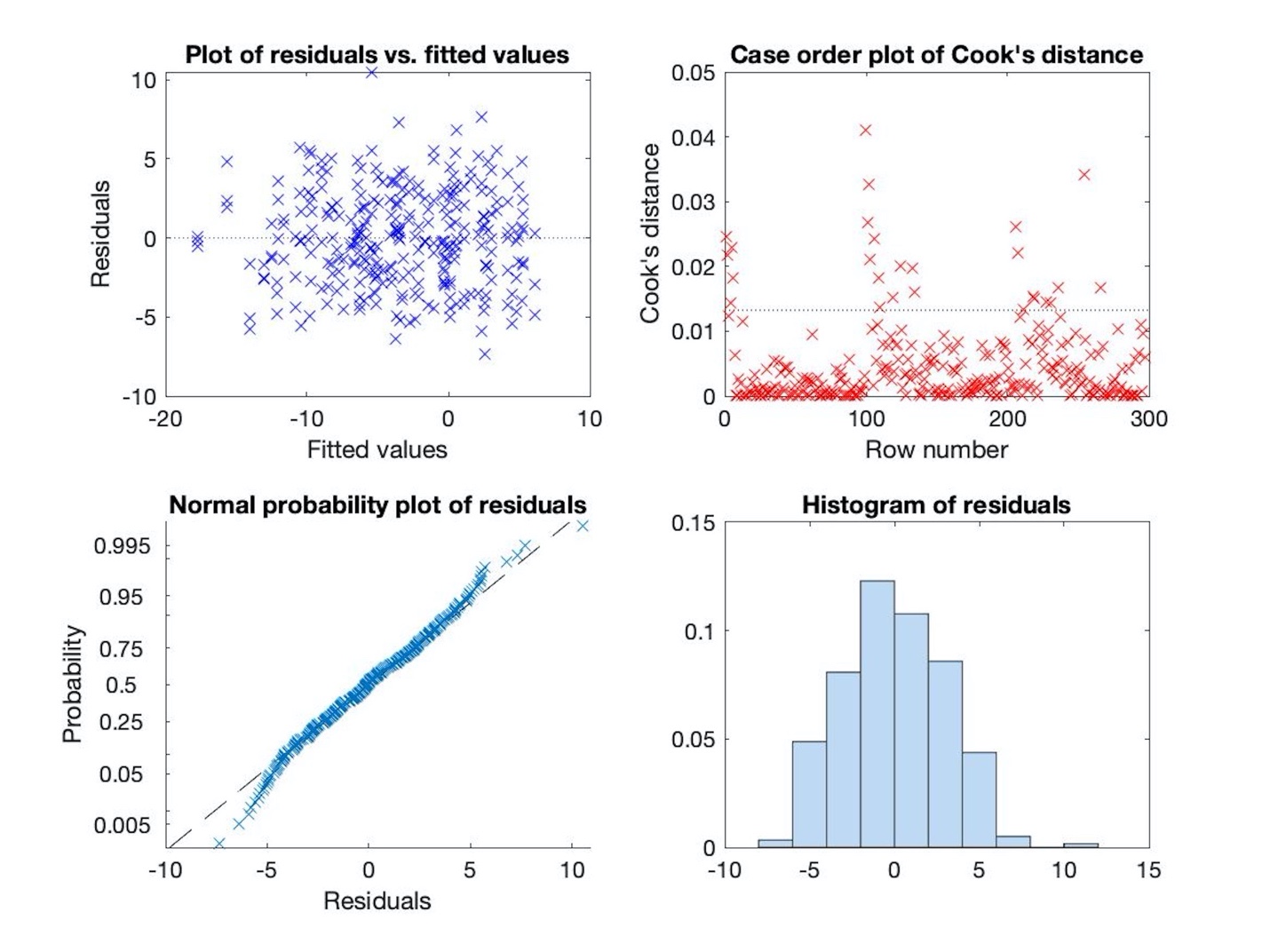


**Figure S6.** Outlier analysis for individual replicates in IDentif.AI AC16 %Cytotoxicity analysis after removing the data points corresponding to OACD combination 15. Row number in Cook’s distance plot corresponds to the combination triplicates in an order specified by the OACD table.


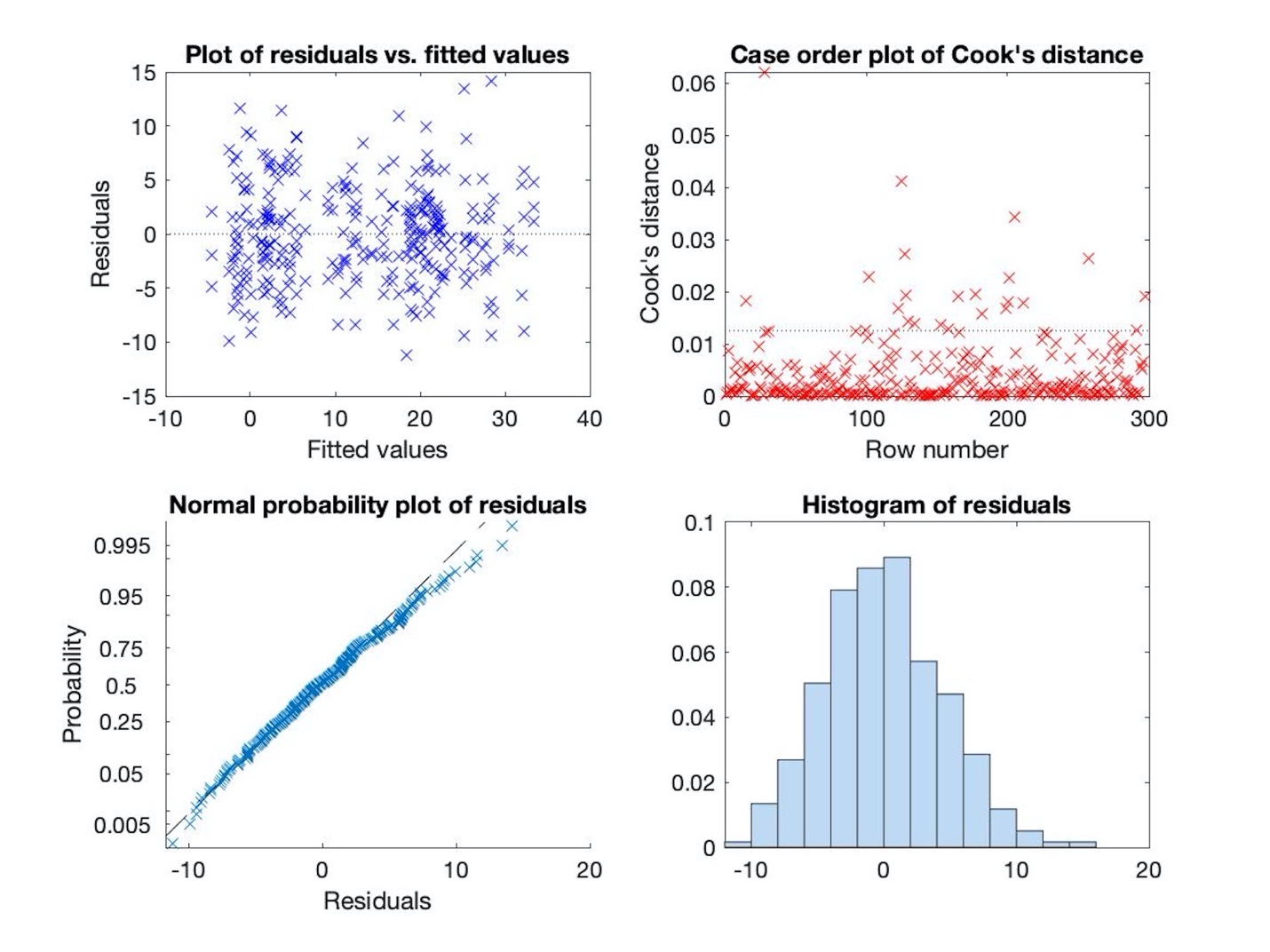


**Figure S7.** Outlier analysis for individual replicates in IDentif.AI THLE-2 %Cytotoxicity analysis after removing the data points corresponding to OACD combination 46. Row number in Cook’s distance plot corresponds to the combination triplicates in an order specified by the OACD table.

**Table S1.** OACD design. 100 combinations for 12 drugs at three different concentration levels (0, 1 and 2). Remdesivir (RDV), favipiravir (FPV), ritonavir (RTV), lopinavir (LPV), ribavirin (RBV), azithromycin (AZT), losartan (LST), dexamethasone (DEX), chloroquine diphosphate (CQ), hydroxychloroquine sulfate (HCQ), oseltamivir phosphate (OSV-P) and teicoplanin (TEC).

| Combo | RDV | FPV | RTV | LPV | RBV | CQ | HCQ | AZT | OSV-P | LST | TEC | DEX |
| --- | --- | --- | --- | --- | --- | --- | --- | --- | --- | --- | --- | --- |
| 1 | 0 | 0 | 0 | 1 | 1 | 0 | 0 | 1 | 0 | 2 | 2 | 0 |
| 2 | 0 | 0 | 0 | 0 | 2 | 0 | 2 | 0 | 2 | 0 | 0 | 1 |
| 3 | 0 | 0 | 1 | 0 | 0 | 2 | 1 | 2 | 0 | 0 | 1 | 0 |
| 4 | 0 | 0 | 2 | 2 | 0 | 1 | 0 | 0 | 1 | 1 | 0 | 0 |
| 5 | 0 | 1 | 2 | 2 | 0 | 0 | 1 | 1 | 2 | 0 | 2 | 2 |
| 6 | 0 | 1 | 2 | 1 | 2 | 1 | 2 | 2 | 2 | 2 | 1 | 0 |
| 7 | 0 | 1 | 0 | 0 | 2 | 2 | 0 | 2 | 1 | 1 | 2 | 2 |
| 8 | 0 | 1 | 1 | 2 | 1 | 2 | 2 | 0 | 0 | 2 | 0 | 2 |
| 9 | 0 | 2 | 1 | 2 | 1 | 0 | 0 | 2 | 2 | 1 | 1 | 1 |
| 10 | 0 | 2 | 1 | 0 | 0 | 1 | 2 | 1 | 1 | 2 | 2 | 1 |
| 11 | 0 | 2 | 2 | 1 | 2 | 2 | 1 | 1 | 0 | 1 | 0 | 1 |
| 12 | 0 | 2 | 0 | 1 | 1 | 1 | 1 | 0 | 1 | 0 | 1 | 2 |
| 13 | 1 | 1 | 1 | 2 | 2 | 1 | 1 | 2 | 1 | 0 | 0 | 1 |
| 14 | 1 | 1 | 1 | 1 | 0 | 1 | 0 | 1 | 0 | 1 | 1 | 2 |
| 15 | 1 | 1 | 2 | 1 | 1 | 0 | 2 | 0 | 1 | 1 | 2 | 1 |
| 16 | 1 | 1 | 0 | 0 | 1 | 2 | 1 | 1 | 2 | 2 | 1 | 1 |
| 17 | 1 | 2 | 0 | 0 | 1 | 1 | 2 | 2 | 0 | 1 | 0 | 0 |
| 18 | 1 | 2 | 0 | 2 | 0 | 2 | 0 | 0 | 0 | 0 | 2 | 1 |
| 19 | 1 | 2 | 1 | 1 | 0 | 0 | 1 | 0 | 2 | 2 | 0 | 0 |
| 20 | 1 | 2 | 2 | 0 | 2 | 0 | 0 | 1 | 1 | 0 | 1 | 0 |
| 21 | 1 | 0 | 2 | 0 | 2 | 1 | 1 | 0 | 0 | 2 | 2 | 2 |
| 22 | 1 | 0 | 2 | 1 | 1 | 2 | 0 | 2 | 2 | 0 | 0 | 2 |
| 23 | 1 | 0 | 0 | 2 | 0 | 0 | 2 | 2 | 1 | 2 | 1 | 2 |
| 24 | 1 | 0 | 1 | 2 | 2 | 2 | 2 | 1 | 2 | 1 | 2 | 0 |
| 25 | 2 | 2 | 2 | 0 | 0 | 2 | 2 | 0 | 2 | 1 | 1 | 2 |
| 26 | 2 | 2 | 2 | 2 | 1 | 2 | 1 | 2 | 1 | 2 | 2 | 0 |
| 27 | 2 | 2 | 0 | 2 | 2 | 1 | 0 | 1 | 2 | 2 | 0 | 2 |
| 28 | 2 | 2 | 1 | 1 | 2 | 0 | 2 | 2 | 0 | 0 | 2 | 2 |
| 29 | 2 | 0 | 1 | 1 | 2 | 2 | 0 | 0 | 1 | 2 | 1 | 1 |
| 30 | 2 | 0 | 1 | 0 | 1 | 0 | 1 | 1 | 1 | 1 | 0 | 2 |
| 31 | 2 | 0 | 2 | 2 | 1 | 1 | 2 | 1 | 0 | 0 | 1 | 1 |
| 32 | 2 | 0 | 0 | 1 | 0 | 1 | 1 | 2 | 2 | 1 | 2 | 1 |
| 33 | 2 | 1 | 0 | 1 | 0 | 2 | 2 | 1 | 1 | 0 | 0 | 0 |
| 34 | 2 | 1 | 0 | 2 | 2 | 0 | 1 | 0 | 0 | 1 | 1 | 0 |
| 35 | 2 | 1 | 1 | 0 | 1 | 1 | 0 | 0 | 2 | 0 | 2 | 0 |
| 36 | 2 | 1 | 2 | 0 | 0 | 0 | 0 | 2 | 0 | 2 | 0 | 1 |
| 37 | 2 | 2 | 2 | 2 | 2 | 2 | 2 | 2 | 2 | 2 | 2 | 2 |
| 38 | 2 | 2 | 2 | 2 | 2 | 0 | 0 | 0 | 0 | 0 | 0 | 0 |
| 39 | 2 | 2 | 2 | 2 | 0 | 2 | 0 | 0 | 0 | 0 | 0 | 0 |
| 40 | 2 | 2 | 2 | 2 | 0 | 0 | 2 | 2 | 2 | 2 | 2 | 2 |
| 41 | 2 | 2 | 2 | 0 | 2 | 2 | 0 | 0 | 0 | 0 | 2 | 2 |
| 42 | 2 | 2 | 2 | 0 | 2 | 0 | 2 | 2 | 2 | 2 | 0 | 0 |
| 43 | 2 | 2 | 2 | 0 | 0 | 2 | 2 | 2 | 2 | 2 | 0 | 0 |
| 44 | 2 | 2 | 2 | 0 | 0 | 0 | 0 | 0 | 0 | 0 | 2 | 2 |
| 45 | 2 | 2 | 0 | 2 | 2 | 2 | 0 | 0 | 2 | 2 | 0 | 0 |
| 46 | 2 | 2 | 0 | 2 | 2 | 0 | 2 | 2 | 0 | 0 | 2 | 2 |
| 47 | 2 | 2 | 0 | 2 | 0 | 2 | 2 | 2 | 0 | 0 | 2 | 2 |
| 48 | 2 | 2 | 0 | 2 | 0 | 0 | 0 | 0 | 2 | 2 | 0 | 0 |
| 49 | 2 | 2 | 0 | 0 | 2 | 2 | 2 | 2 | 0 | 0 | 0 | 0 |
| 50 | 2 | 2 | 0 | 0 | 2 | 0 | 0 | 0 | 2 | 2 | 2 | 2 |
| 51 | 2 | 2 | 0 | 0 | 0 | 2 | 0 | 0 | 2 | 2 | 2 | 2 |
| 52 | 2 | 2 | 0 | 0 | 0 | 0 | 2 | 2 | 0 | 0 | 0 | 0 |
| 53 | 2 | 0 | 2 | 2 | 2 | 2 | 0 | 2 | 0 | 2 | 0 | 2 |
| 54 | 2 | 0 | 2 | 2 | 2 | 0 | 2 | 0 | 2 | 0 | 2 | 0 |
| 55 | 2 | 0 | 2 | 2 | 0 | 2 | 2 | 0 | 2 | 0 | 2 | 0 |
| 56 | 2 | 0 | 2 | 2 | 0 | 0 | 0 | 2 | 0 | 2 | 0 | 2 |
| 57 | 2 | 0 | 2 | 0 | 2 | 2 | 2 | 0 | 2 | 0 | 0 | 2 |
| 58 | 2 | 0 | 2 | 0 | 2 | 0 | 0 | 2 | 0 | 2 | 2 | 0 |
| 59 | 2 | 0 | 2 | 0 | 0 | 2 | 0 | 2 | 0 | 2 | 2 | 0 |
| 60 | 2 | 0 | 2 | 0 | 0 | 0 | 2 | 0 | 2 | 0 | 0 | 2 |
| 61 | 2 | 0 | 0 | 2 | 2 | 2 | 2 | 0 | 0 | 2 | 2 | 0 |
| 62 | 2 | 0 | 0 | 2 | 2 | 0 | 0 | 2 | 2 | 0 | 0 | 2 |
| 63 | 2 | 0 | 0 | 2 | 0 | 2 | 0 | 2 | 2 | 0 | 0 | 2 |
| 64 | 2 | 0 | 0 | 2 | 0 | 0 | 2 | 0 | 0 | 2 | 2 | 0 |
| 65 | 2 | 0 | 0 | 0 | 2 | 2 | 0 | 2 | 2 | 0 | 2 | 0 |
| 66 | 2 | 0 | 0 | 0 | 2 | 0 | 2 | 0 | 0 | 2 | 0 | 2 |
| 67 | 2 | 0 | 0 | 0 | 0 | 2 | 2 | 0 | 0 | 2 | 0 | 2 |
| 68 | 2 | 0 | 0 | 0 | 0 | 0 | 0 | 2 | 2 | 0 | 2 | 0 |
| 69 | 0 | 2 | 2 | 2 | 2 | 2 | 0 | 2 | 2 | 0 | 2 | 0 |
| 70 | 0 | 2 | 2 | 2 | 2 | 0 | 2 | 0 | 0 | 2 | 0 | 2 |
| 71 | 0 | 2 | 2 | 2 | 0 | 2 | 2 | 0 | 0 | 2 | 0 | 2 |
| 72 | 0 | 2 | 2 | 2 | 0 | 0 | 0 | 2 | 2 | 0 | 2 | 0 |
| 73 | 0 | 2 | 2 | 0 | 2 | 2 | 2 | 0 | 0 | 2 | 2 | 0 |
| 74 | 0 | 2 | 2 | 0 | 2 | 0 | 0 | 2 | 2 | 0 | 0 | 2 |
| 75 | 0 | 2 | 2 | 0 | 0 | 2 | 0 | 2 | 2 | 0 | 0 | 2 |
| 76 | 0 | 2 | 2 | 0 | 0 | 0 | 2 | 0 | 0 | 2 | 2 | 0 |
| 77 | 0 | 2 | 0 | 2 | 2 | 2 | 2 | 0 | 2 | 0 | 0 | 2 |
| 78 | 0 | 2 | 0 | 2 | 2 | 0 | 0 | 2 | 0 | 2 | 2 | 0 |
| 79 | 0 | 2 | 0 | 2 | 0 | 2 | 0 | 2 | 0 | 2 | 2 | 0 |
| 80 | 0 | 2 | 0 | 2 | 0 | 0 | 2 | 0 | 2 | 0 | 0 | 2 |
| 81 | 0 | 2 | 0 | 0 | 2 | 2 | 0 | 2 | 0 | 2 | 0 | 2 |
| 82 | 0 | 2 | 0 | 0 | 2 | 0 | 2 | 0 | 2 | 0 | 2 | 0 |
| 83 | 0 | 2 | 0 | 0 | 0 | 2 | 2 | 0 | 2 | 0 | 2 | 0 |
| 84 | 0 | 2 | 0 | 0 | 0 | 0 | 0 | 2 | 0 | 2 | 0 | 2 |
| 85 | 0 | 0 | 2 | 2 | 2 | 2 | 2 | 2 | 0 | 0 | 0 | 0 |
| 86 | 0 | 0 | 2 | 2 | 2 | 0 | 0 | 0 | 2 | 2 | 2 | 2 |
| 87 | 0 | 0 | 2 | 2 | 0 | 2 | 0 | 0 | 2 | 2 | 2 | 2 |
| 88 | 0 | 0 | 2 | 2 | 0 | 0 | 2 | 2 | 0 | 0 | 0 | 0 |
| 89 | 0 | 0 | 2 | 0 | 2 | 2 | 0 | 0 | 2 | 2 | 0 | 0 |
| 90 | 0 | 0 | 2 | 0 | 2 | 0 | 2 | 2 | 0 | 0 | 2 | 2 |
| 91 | 0 | 0 | 2 | 0 | 0 | 2 | 2 | 2 | 0 | 0 | 2 | 2 |
| 92 | 0 | 0 | 2 | 0 | 0 | 0 | 0 | 0 | 2 | 2 | 0 | 0 |
| 93 | 0 | 0 | 0 | 2 | 2 | 2 | 0 | 0 | 0 | 0 | 2 | 2 |
| 94 | 0 | 0 | 0 | 2 | 2 | 0 | 2 | 2 | 2 | 2 | 0 | 0 |
| 95 | 0 | 0 | 0 | 2 | 0 | 2 | 2 | 2 | 2 | 2 | 0 | 0 |
| 96 | 0 | 0 | 0 | 2 | 0 | 0 | 0 | 0 | 0 | 0 | 2 | 2 |
| 97 | 0 | 0 | 0 | 0 | 2 | 2 | 2 | 2 | 2 | 2 | 2 | 2 |
| 98 | 0 | 0 | 0 | 0 | 2 | 0 | 0 | 0 | 0 | 0 | 0 | 0 |
| 99 | 0 | 0 | 0 | 0 | 0 | 2 | 0 | 0 | 0 | 0 | 0 | 0 |
| 100 | 0 | 0 | 0 | 0 | 0 | 0 | 2 | 2 | 2 | 2 | 2 | 2 |

**Table S2.** Estimates and significance of IDentif.AI–designed %Inhibition coefficients (12 drugs at three levels). Remdesivir (RDV), favipiravir (FPV), ritonavir (RTV), lopinavir (LPV), ribavirin (RBV), azithromycin (AZT), losartan (LST), dexamethasone (DEX), chloroquine diphosphate (CQ), hydroxychloroquine sulfate (HCQ), oseltamivir phosphate (OSV-P) and teicoplanin (TEC). Statistical analyses were done using sum of squares F-test. **P* < 0.05, ***P* < 0.01 and ****P* < 0.001.

|  | **Estimate** | **Significance** |
| --- | --- | --- |
| Intercept | 3.2672 | ** |
| RDV | -91.635 | *** |
| FPV | -0.6124 | ** |
| RTV | -1.0895 |  |
| LPV | -3.9092 | ** |
| RBV | 16.627 | *** |
| CQ | -23.15 |  |
| HCQ | -2.1163 |  |
| AZT | -51.533 |  |
| OSV-P | -168.36 |  |
| LST | -1049.3 | *** |
| TEC | -1.6679 |  |
| DEX | -28.936 |  |
| RDV:FPV | -0.15529 | * |
| RDV:LPV | 21.919 | *** |
| RDV:RBV | 3.768 | ** |
| RDV:CQ | -41.376 | ** |
| RDV:AZT | 496.18 | *** |
| RDV:LST | -784.47 | *** |
| FPV:HCQ | -1.0398 | *** |
| FPV:AZT | 5.8365 |  |
| FPV:OSV-P | 43.69 | *** |
| RTV:LPV | 14.412 | *** |
| RTV:RBV | 3.5581 | ** |
| RTV:AZT | -350.24 | *** |
| RTV:OSV-P | -621.99 | *** |
| RTV:LST | 332.86 | * |
| RTV:DEX | 267.6 | *** |
| LPV:RBV | 1.0079 |  |
| LPV:LST | -413.68 | *** |
| RBV:HCQ | -3.7614 |  |
| CQ:HCQ | 51.445 | * |
| CQ:LST | 1453.6 | * |
| CQ:DEX | 601.19 | ** |
| HCQ:OSV-P | 1649.9 | *** |
| HCQ:LST | 1955.1 | *** |
| HCQ:TEC | -9.6429 |  |
| HCQ:DEX | -409.56 | *** |
| AZT:OSV-P | -44632 | *** |
| AZT:LST | 17207 | ** |
| AZT:TEC | 375.24 | ** |
| RDV^2^ | 125.73 | *** |
| FPV^2^ | 0.016541 | ** |
| RBV^2^ | -10.335 | *** |
| LST^2^ | 35487 | ** |
| Adj R^2^ (IDentif.AI) | 0.898 | |

**Table S3.** Estimates and significance of IDentif.AI–designed Vero E6 %Cytotoxicity coefficients (12 drugs at three levels). Remdesivir (RDV), favipiravir (FPV), ritonavir (RTV), lopinavir (LPV), ribavirin (RBV), azithromycin (AZT), losartan (LST), dexamethasone (DEX), chloroquine diphosphate (CQ), hydroxychloroquine sulfate (HCQ), oseltamivir phosphate (OSV-P) and teicoplanin (TEC). Statistical analyses were done using sum of squares F-test. **P* < 0.05, ***P* < 0.01 and ****P* < 0.001.

|  | **Estimate** | **Significance** |
| --- | --- | --- |
| Intercept | 1.8634 |  |
| RDV | -0.3778 |  |
| FPV | 0.030642 |  |
| RTV | -2.8728 |  |
| LPV | -0.79633 |  |
| RBV | 10.416 | ** |
| HCQ | 29.709 | * |
| AZT | 408.84 | * |
| OSV-P | -77.444 |  |
| LST | -151.62 | * |
| DEX | -27.802 |  |
| RDV:LPV | 2.2405 |  |
| RDV:HCQ | -12.364 | * |
| RDV:LST | 222.07 | * |
| FPV:LPV | -0.065286 | * |
| RTV:OSV-P | 336.47 | * |
| RBV:HCQ | 7.2941 | *** |
| OSV-P:DEX | -3231.9 |  |
| RBV^2^ | -6.3945 | ** |
| HCQ^2^ | -47.634 | * |
| AZT^2^ | -13103 | * |
| Adjusted R^2^ (IDentif.AI) | 0.145 | |

**Table S4.** Estimates and significance of IDentif.AI–designed AC16 %Cytotoxicity coefficients (12 drugs at three levels). Remdesivir (RDV), favipiravir (FPV), ritonavir (RTV), lopinavir (LPV), ribavirin (RBV), azithromycin (AZT), losartan (LST), dexamethasone (DEX), chloroquine diphosphate (CQ), hydroxychloroquine sulfate (HCQ), oseltamivir phosphate (OSV-P) and teicoplanin (TEC). Statistical analyses were done using sum of squares F-test. **P* < 0.05, ***P* < 0.01 and ****P* < 0.001.

|  | **Estimate** | | **Significance** |
| --- | --- | --- | --- |
| Intercept | -2.5613 | | * |
| RDV | -23.613 | | *** |
| FPV | 0.24715 | |  |
| RTV | -0.41271 | |  |
| LPV | -2.9274 | |  |
| RBV | 16.916 | | *** |
| CQ | 10.493 | |  |
| HCQ | 93.283 | | *** |
| AZT | 518.99 | | ** |
| OVS-P | 475.72 | |  |
| LST | -2026.5 | | *** |
| TEC | 4.2827 | |  |
| DEX | -877.45 | | *** |
| RDV:TEC | 17.79 | | *** |
| RDV:DEX | 734.04 | | *** |
| FPV:LPV | -0.096566 | | * |
| FPV:RBV | 0.071665 | | *** |
| FPV:HCQ | -0.85933 | | *** |
| FPV:AZT | 5.424 | | * |
| FPV:OSV-P | -22.303 | | *** |
| FPV:TEC | -0.90248 | | *** |
| RTV:LPV | -5.1743 | | ** |
| RTV:AZT | 440.87 | | *** |
| RTV:OSV-P | 715.3 | | *** |
| RTV:LST | 762.96 | | *** |
| LPV:HCQ | -15.607 | | *** |
| LPV:AZT | -175.47 | | *** |
| LPV:TEC | -9.175 | | *** |
| RBV:DEX | -22.637 | | ** |
| CQ:HCQ | -32.992 | | ** |
| CQ:DEX | 293.28 | | ** |
| HCQ:AZT | -1286.7 | | *** |
| HCQ:TEC | -44.507 | | *** |
| HCQ:DEX | -212.07 | | * |
| AZT:TEC | 282.95 | | ** |
| AZT:DEX | 11643 | | *** |
| OSV-P:TEC | 2380.5 | | *** |
| OSV-P:DEX | 1599 | |  |
| LST:DEX | -22970 | | *** |
| TEC:DEX | 362.07 | | *** |
| FPV^2^ | 0.016801 | | *** |
| RTV^2^ | -15.444 | | *** |
| LPV^2^ | 9.6639 | | *** |
| RBV^2^ | -11.148 | | *** |
| HCQ^2^ | -25.664 | | * |
| AZT^2^ | -25628 | | *** |
| OSV-P^2^ | -92834 | | *** |
| LST^2^ | 1.0952e+05 | | *** |
| TEC^2^ | -13.035 | | *** |
| DEX^2^ | 5911.8 | | *** |
| Adjusted R^2^ (IDentif.AI) | | 0.708 | |

**Table S5.** Estimates and significance of IDentif.AI–designed THLE-2 %Cytotoxicity coefficients (12 drugs at three levels). Remdesivir (RDV), favipiravir (FPV), ritonavir (RTV), lopinavir (LPV), ribavirin (RBV), azithromycin (AZT), losartan (LST), dexamethasone (DEX), chloroquine diphosphate (CQ), hydroxychloroquine sulfate (HCQ), oseltamivir phosphate (OSV-P) and teicoplanin (TEC). Statistical analyses were done using sum of squares F-test. **P* < 0.05, ***P* < 0.01 and *** *P*< 0.001.

|  | **Estimate** | **Significance** |
| --- | --- | --- |
| Intercept | 0.92098 |  |
| RDV | 151.43 | *** |
| FPV | -2.0009 | *** |
| RTV | 69.644 | *** |
| LPV | -10.976 | *** |
| RBV | -6.4728 |  |
| CQ | -185.42 | *** |
| HCQ | -37.801 | *** |
| AZT | -250.23 | ** |
| OVS-P | -159.58 |  |
| LST | 1326.2 | *** |
| TEC | -56.531 | *** |
| DEX | 491.38 | ** |
| RDV:RTV | -20.069 | *** |
| RDV:CQ | -29.039 | ** |
| RDV:LST | -2233 | *** |
| RDV:TEC | -24.426 | *** |
| FPV:RTV | -0.54238 | *** |
| FPV:LPV | 0.21444 | ** |
| FPV:HCQ | 1.8627 | *** |
| FPV:OVS-P | 50.553 | *** |
| FPV:LST | -14.63 | ** |
| FPV:DEX | 10.464 | *** |
| RTV:LPV | -5.2925 | * |
| RTV:HCQ | 23.603 | *** |
| RTV:AZT | 410.04 | *** |
| RTV:OVS-P | -1958.5 | *** |
| RTV:LST | -965.21 | *** |
| RTV:TEC | 23.483 | *** |
| RTV:DEX | -694.46 | *** |
| LPV:RBV | 0.96287 | * |
| LPV:OVS-P | -739.95 | *** |
| LPV:LST | 1048.7 | *** |
| LPV:TEC | 16.48 | *** |
| LPV:DEX | -125.53 | ** |
| RBV:AZT | 52.497 | * |
| RBV:OVS-P | -90.632 | * |
| HCQ:LST | 1524.7 | *** |
| HCQ:TEC | 12.626 | * |
| HCQ:DEX | -354.21 | ** |
| AZT:LST | 25773 | *** |
| AZT:DEX | -5870.8 | ** |
| OVS-P:LST | -1.388e+05 | *** |
| OVS-P:TEC | 1205.8 | ** |
| LST:DEX | -8046.1 | * |
| TEC:DEX | 790.98 | *** |
| DEX | -88.422 | ** |
| FPV^2^ | 0.025596 | *** |
| RTV^2^ | -20.216 | *** |
| RBV^2^ | 4.41 | * |
| CQ^2^ | 1377.6 | *** |
| HCQ^2^ | -37.733 |  |
| OVS-P^2^ | 1.1007e+05 | *** |
| DEX^2^ | -5830.6 | ** |
| Adjusted R^2^ (IDentif.AI) | 0.809 | |
